# Spatial multi‐omics unveils sphingolipid metabolic reprogramming within the retinal pathological niche

**DOI:** 10.1002/imt2.70163

**Published:** 2026-08-20

**Authors:** Yunhong Shi, Jinpei Lin, Yi Wu, Shengsong Xu, Naiyuan Zhang, Yiji Pan, Anli Ren, Ying Fang, Yu Huang, Xiayin Zhang, Zhuoting Zhu, Yehong Zhuo, Honghua Yu

**Affiliations:** ^1^ Guangdong Eye Institute, Department of Ophthalmology, Guangdong Provincial People's Hospital (Guangdong Academy of Medical Sciences) Southern Medical University Guangzhou China; ^2^ State Key Laboratory of Ophthalmology, Zhongshan Ophthalmic Center Sun Yat‐sen University, Guangdong Provincial Key Laboratory of Ophthalmology Visual Science Guangzhou China; ^3^ College of Life Sciences University of Chinese Academy of Sciences Beijing China; ^4^ Analysis Center of Agrobiology and Environmental Sciences Zhejiang University Hangzhou China; ^5^ Shenzhen Eye Hospital, Shenzhen Eye Medical Center Southern Medical University Shenzhen China; ^6^ Department of Eye and Vision Sciences, Institute of Life Course and Medical Sciences University of Liverpool Liverpool UK; ^7^ Singapore Eye Research Institute Singapore National Eye Center Singapore Singapore; ^8^ Centre for Eye Research Australia, University of Melbourne, Department of Surgery (Ophthalmology) University of Melbourne Melbourne Australia; ^9^ Joint Shantou International Eye Center of Shantou University and The Chinese University of Hong Kong Shantou China

**Keywords:** immunometabolic remodeling, retinal ischemia‐reperfusion, spatial metabolomics, spatial transcriptomics, *Trem2* ^+^ microglia

## Abstract

Retinal ischemia‐reperfusion (RIR) injury is a central mechanism underlying irreversible vision loss in glaucoma and other retinal diseases, yet the spatial organization of the pathogenic microenvironment remains poorly understood. Here, we constructed a high‐resolution, whole‐eye spatial multi‐omics atlas integrating Stereo‐seq transcriptomics and MALDI‐MSI‐based metabolomics to capture the early molecular events in the RIR mouse model. Spatially, retinal ganglion cell (RGC) interactions with surrounding cells were markedly reduced after injury, whereas immune–glial interactions were enhanced, revealing a shift from a neuron‐centered to an immunometabolic state. Within the ganglion cell layer (GCL), the primary pathological locus, we identified *Trem2* ^*+*^ microglia that expand in situ, establish close proximity to degenerating RGCs, and exhibit strong spatial association with dysregulated sphingolipid metabolism. Mechanistically, using *Trem2* knockout mice and microglia‐specific *Trem2* siRNA knockdown, we demonstrate that TREM2 directly binds to SPTLC2, the rate‐limiting enzyme of de novo sphingolipid biosynthesis, driving ceramide‐centric metabolic reprogramming that modulates the AKT‐mTOR signaling axis and amplifies inflammatory activation. Pharmacological inhibition of serine palmitoyl transferase (SPT) with myriocin reverses this cascade, protecting RGCs. Multi‐omics integration of human glaucoma aqueous humor datasets further reveals conserved upregulation of sphingolipid biosynthetic enzymes, sphingolipid metabolites, and lipid‐sensing immune effectors, offering preliminary translational clues. Collectively, our findings reveal that spatially defined immunometabolic remodeling—in which *Trem2* ^*+*^ microglia are central—converges on sphingolipid metabolism as a druggable regulatory axis, with the TREM2‐SPTLC2 interface thus emerging as a new therapeutic opportunity for retinal neurodegenerative diseases.

## Highlights

- Spatial multi‐omics maps the pathogenic niche in retinal ischemia‐reperfusion (RIR), revealing a shift from neuronal to immunometabolic networks in the inner retina.
- *Trem2* ^*+*^ microglial subpopulation expands in the ganglion cell layer and co‐localizes with degenerating retinal ganglion cells within this niche.
- TREM2 interacts with SPTLC2 and is spatially associated with local sphingolipid metabolic remodeling within the retinal pathological niche.
- Pharmacological SPT inhibition with myriocin reverses microglial inflammation and protects retinal ganglion cells, identifying the TREM2‐SPTLC2 interface as a druggable target.

## Graphical abstract

Spatial multi‐omics of the mouse ocular globe after retinal ischemia‐reperfusion (RIR) injury identifies the ganglion cell layer (GCL) as an immunometabolic niche where *Trem2* ^+^ microglia closely associate with stressed retinal ganglion cells (RGCs) through *Trem2‐Apoe* signaling. Genetic deletion of *Trem2* attenuates microglial inflammation and protects RGCs. Spatial metabolomics further reveals that *Trem2* is associated with the accumulation of sphingolipid metabolites within the GCL. Spatial metabolomics reveals that TREM2‐expressing microglia co‐localize with altered sphingolipid ion signatures. Mechanistically, TREM2 interacts with SPTLC2, suggesting a potential regulatory interface that correlates with inflammatory activation. These findings suggest a TREM2‐SPTLC2‐sphingolipid axis as a spatially confined, potentially druggable pathogenic mechanism in RIR injury.

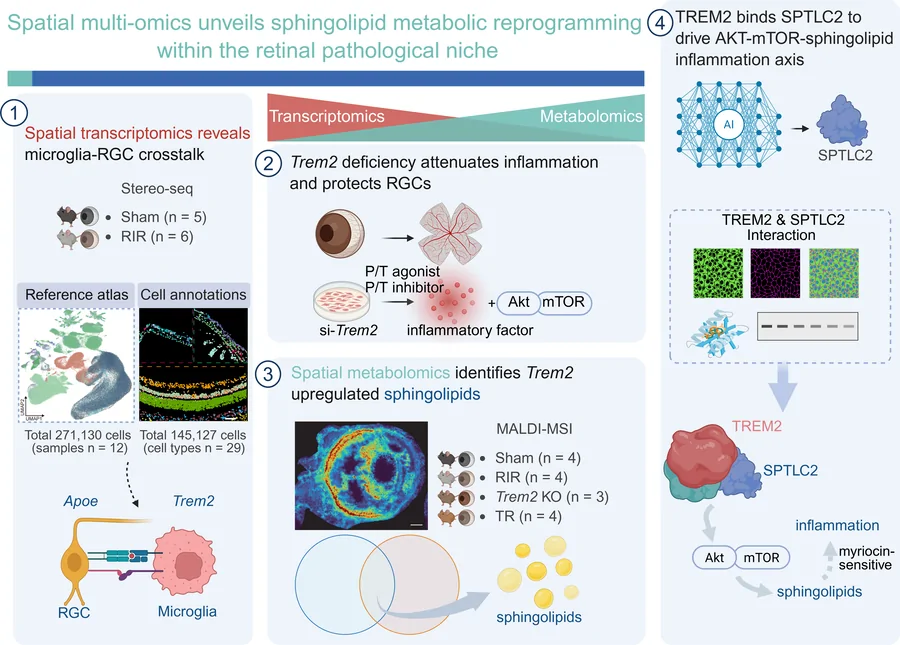

## INTRODUCTION

Retinal ischemia‐reperfusion (RIR) is a core pathological mechanism underlying irreversible vision loss following glaucoma (especially acute angle‐closure glaucoma) [1], various ophthalmic emergencies (e.g., retinal artery occlusion, central retinal vein occlusion, ocular ischemic syndrome) [2], and systemic ischemic events (e.g., post‐cardiac arrest resuscitation, carotid artery surgery) [3]. Interruption of blood supply leads to severe hypoxia and energy depletion in the retina; upon reperfusion, bursts of excessive reactive oxygen species (ROS), mitochondrial permeability transition, calcium overload, and inflammatory cascades further exacerbate neuronal death, causing more extensive damage than ischemia alone [4, 5, 6]. The ultimate consequence of RIR injury is the rapid, progressive loss of RGCs and their axons, clinically manifesting as visual field defects and reduced visual acuity, with no current therapy capable of effectively reversing injured neurons [7]. RIR injury does not affect the entire retina uniformly but exhibits pronounced spatial dependence. The retina possesses a highly ordered laminar architecture, in which neurons and glial cells across its ten anatomical layers achieve phototransduction, synaptic integration, and microenvironmental homeostasis through precisely organized spatial relationships [8]. This spatial architecture simultaneously dictates regional metabolic demands and injury susceptibility. The retina's oxygen consumption rate per unit weight exceeds that of the brain, and RGCs are highly vulnerable to disturbances in energy metabolism [9, 10]. Metabolomic studies using homogenized tissues have revealed metabolic abnormalities in RIR or glaucoma, including mitochondrial damage, excessive ROS, glycogen depletion, and dysregulated anaerobic glycolysis [11, 12, 13]; these changes often precede overt neuronal degeneration, suggesting that metabolic dysfunction is an early driving event [14, 15]. However, conventional homogenization‐based omics approaches disrupt the retina's intrinsic layering, obscuring the spatial origins of metabolites, interlayer heterogeneity of metabolic states, and cell‐type‐specific contributions [16, 17, 18]. Consequently, we are unable to decipher how metabolic stress is initiated within specific RGCs microenvironments or how lipid signaling pathways become dysregulated within particular cellular neighborhoods.

To dissect molecular events while preserving native tissue architecture, spatial transcriptomics (ST) has emerged, enabling in situ characterization of transcriptional programs across different regions by retaining spatial context information [19, 20]. In recent years, spatial multi‐omics integrating ST, spatial metabolomics (SM), spatial proteomics, and others has become a frontier in neurodegenerative disease research [21, 22]. The integration of multi‐omics with artificial intelligence‐driven approaches has emerged as a core developmental direction, while spatial omics has entered the “micro‐resolution era,” where subcellular analysis, three‐dimensional spatial omics, and multi‐omics fusion are forming an irreversible trend [23, 24, 25]. On the technological front, Stereo‐seq V2 achieves subcellular‐resolution whole‐transcriptome in situ detection in Formalin‐Fixed, Paraffin‐Embedded samples [26]; iPEX technology, via expanded tissue coupled with MALDI mass spectrometry imaging, enables micrometer‐depth spatial proteomic analysis [27]. These breakthroughs make it possible to systematically map the molecular landscape of distinct retinal layers and microregions following RIR in the native tissue context.

In this study, we used a RIR model to capture early molecular and cellular changes induced by acute ocular hypertension and constructed a high‐resolution spatial multi‐omics atlas of the entire mouse eye globe. By integrating transcriptomic and metabolomic data within their native spatial context, we identified the retinal inner layers as the primary pathological region and uncovered focal accumulation of *Trem2* ^*+*^ microglia accompanied by cell‐type‐specific sphingolipid dysregulation. Mechanistic investigations further demonstrated that *Trem2* regulates sphingolipid metabolic reprogramming through its interaction with SPTLC2. These findings provide new spatial and metabolic insights into early glaucomatous pathology and support the development of metabolically targeted neuroprotective strategies.

## RESULTS

### High‐resolution ST delineates region‐specific molecular architecture in the RIR ocular globes

**Figure 1:**
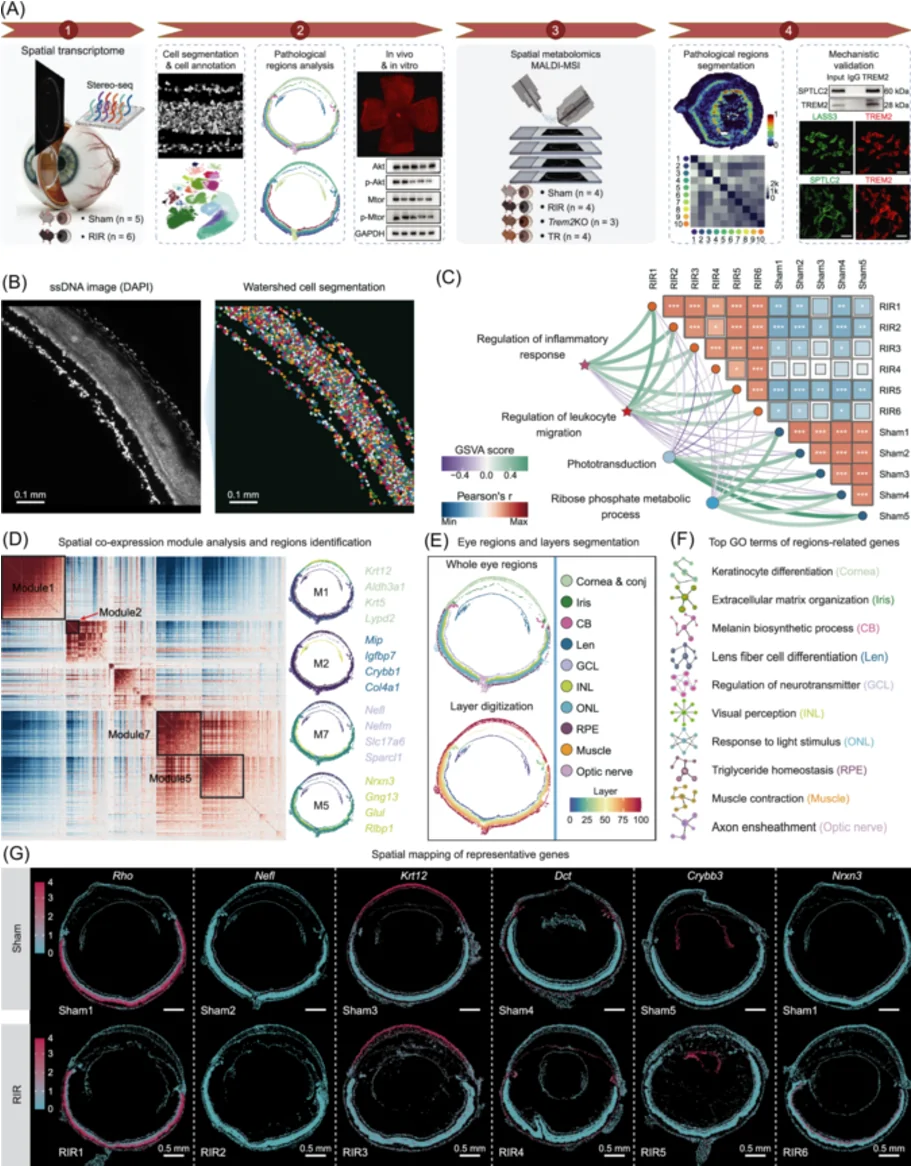
Single‐cell resolution spatial transcriptomics reveals region‐specific molecular architecture of the RIR ocular globe. (A) Experimental design and analysis workflow encompassing spatial transcriptomics, spatial metabolomics, and in vitro validation. (B) Example of watershed‐based cell segmentation performed on nuclear staining (single‐stranded DNA). Left: nuclear staining (single‐stranded DNA); right: segmented cell boundaries (mask). (C) Reproducibility and functional coherence across samples. Left: chord diagram of GSVA scores for ocular function pathways; right: heatmap of sample‐to‐sample correlations. (D) Utilization of spatial co‐expressed genes to redefine the ocular region. Heatmap displays hotspot spatial co‐expression modules associated with specific ocular structures. (E) Anatomical regional division of the eyeball (upper) and the digital division of the eyeball (lower). Posterior segment: GCL, 1–25; INL, 26–50; ONL, 51–75; RPE & muscle, 76–100. Anterior segment: len, 1–25; ciliary body & iris, 26–50; cornea & conjunctiva, 51–100. (F) Left: schematic of ocular region‐specific gene modules; Right: GO enrichment analysis validating their functional correspondence to each anatomical region. (G) Spatial in situ distribution of canonical marker genes. *Rho* (rod photoreceptors) in ONL, *Nefl* (ganglion cells) in GCL, *Krt12* (corneal epithelium) in cornea, *Dct* (melanocytes) in ciliary body/iris, *Crybb3* in len, and *Nrxn3* (amacrine cells) in INL. CB, Ciliary Body; Cornea & conj, Cornea and conjunctiva; GCL, Ganglion cell layer; GO, Gene Ontology; GSEA, Gene Set Enrichment Analysis; GSVA, Gene Set Variation Analysis score; INL, Inner nuclear layer; Muscle, Extraocular Muscle; ONL, Outer nuclear layer; RIR, Retinal Ischemia‐Reperfusion; RPE, Retinal pigment epithelium.

To systematically map the spatial molecular dynamics of RIR, we performed Stereo‐seq on intact mouse eye globes from five Sham and six 72‐h post‐reperfusion RIR samples, combined with SM and in vivo/in vitro validation (Figure 1A). Robust cell segmentation is critical for constructing single‐cell resolved spatial transcriptomic atlases. This proves particularly challenging in retinal tissue due to its dense cellular architecture and heterogeneous morphometric features across neuronal subtypes. High‐resolution ssDNA imaging coupled with computational cell segmentation revealed preserved retinal laminar architecture while enabling precise alignment of transcriptomic data to anatomical subregions (Figure 1B). Inter‐group transcript count analysis demonstrated high intra‐group correlation coefficients, confirming minimal technical variability and robust reproducibility across biological replicates (Figure 1C).

Through spatial co‐expression module analysis, we identified multiple gene modules that were significantly associated with anatomical structures from the mouse ocular spatial transcriptomic data (Figure 1D). Based on these findings, we further curated 10 gene sets corresponding to the classic anatomical structures of the eye (Figure 1E), that is, Cornea & conj, Iris, CB (ciliary body), Len, GCL (ganglion cell layer), INL (inner nuclear layer), ONL (outer nuclear layer), RPE (retinal pigment epithelium), Optic nerve, and Muscle (Figure S1C,D). Digital reconstruction of ocular topology through graph‐based clustering recapitulated known anatomical boundaries while revealing novel subregional molecular heterogeneity (Table S1).

As exemplified by the corneal and conjunctival regions, these compartments exhibited elevated expression of *Krt12, Aldh3a1, Krt5, Krt6a*, and *Col17a1*, while the GCL exhibited marked enrichment of *Calb2, Sncg, Nefm*, and *Nefl*. Gene Ontology (GO) enrichment of module‐specific markers highlighted functional specialization across regions, with GCL‐enriched genes implicating axon guidance and synaptic signaling, while ONL‐associated modules were dominated by phototransduction and retinal metabolism pathways (Figure 1F). Spatial in situ cellular mapping resolved layer‐specific expression of canonical markers, with *Rho* (rod photoreceptors) localizing to the ONL, *Nefl* (ganglion cells) to the GCL, and *Krt12* (corneal epithelium) to the cornea, among others (Figure 1G).

### Spatial reorganization of multiple cell types following RIR

**Figure 2:**
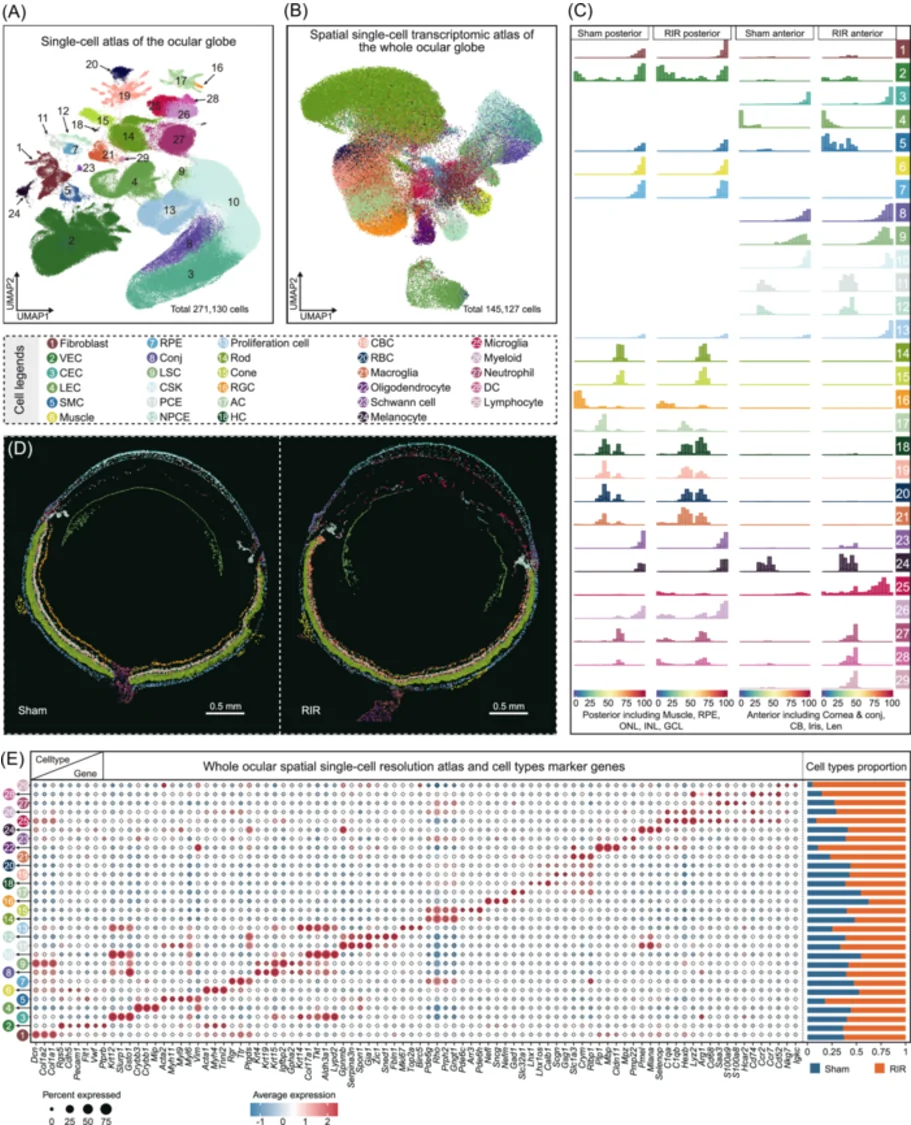
Single‐cell spatial cartography of the Sham and RIR ocular globes. (A) UMAP plot of ocular single cells, colored by 27 cell types. (B) UMAP plot of spatial transcriptomics data from the whole eyeball, featuring 29 cell types. (C) Histograms delineate the anterior‐to‐posterior spatial density distribution dynamics of diverse cell types in Sham and RIR groups. The anterior segment is anatomically defined as comprising the cornea, iris, len, and ciliary body (1–100 means Len → Cornea), while the posterior segment encompasses the retina, choroid, and sclera (1–100 means GCL → RPE). (D) Spatial in situ mapping of single‐cell transcriptomics in mouse ocular globes. The left panel shows the Sham group, and the right panel shows the RIR (glaucoma model) group. Each dot represents a single cell, colored according to 29 distinct cell types. (E) Combined dot plot and stacked bar chart showing cell type annotation validation and compositional changes in whole‐eye spatial transcriptomics. Left: dot plot with 29 cell types (*y*‑axis) and a panel of classic/marker genes (*x*‐axis) used to verify the accuracy of cell types annotation. Right: stacked bar chart illustrating the proportional composition of each cell type in the Sham and RIR groups, based on whole‐eye spatial transcriptomic data. Cell type color codes are consistent across all subpanels (A–E), with the color legend for the 29 cell types positioned above panel. GCL, Ganglion Cell Layer; RIR, Retinal Ischemia‐Reperfusion; RPE, Retinal Pigment Epithelium; UMAP, Uniform Manifold Approximation and Projection.

We integrated 12 publicly available single‐cell RNA sequencing (scRNA‐seq) datasets to construct a reference atlas for spatially resolved cell characterization (Figure S1A, Table S2). This reference dataset comprises 271,130 cells and identified 27 transcriptionally distinct cell clusters (Figures 2A, S1B). By incorporating spatial region‐specific annotations, we ultimately built a single‐cell‐resolution spatial transcriptomic atlas for RIR, encompassing 29 cell populations (Figures 2B, S1C,D). These include: vascular endothelial cell (VEC), smooth muscle cell (SMC), Schwann cell, RPE, rod photoreceptor (Rod), RGC, rod bipolar cell (RBC), proliferating cell (including transit‐amplifying cell), pigmented ciliary epithelium (PCE), non‐pigmented ciliary epithelium (NPCE), neutrophil, myeloid cell, extraocular muscle cell, microglia, melanocyte, macroglia, lymphocyte, limbal stem cell (LSC), len epithelial cell (LEC), horizontal cell (HC), fibroblast, dendritic cell (DC), corneal stromal keratocyte (CSK), conjunctival cell (Conj), cone photoreceptor (Cone), corneal epithelial cell (CEC), cone bipolar cell (CBC), oligodendrocyte, and amacrine cell (AC). Cell type annotation was based on known marker genes (Figure 2E). Their unique spatial expression patterns validated the high fidelity of our ST‐based cellular characterization (Figure 2D). Furthermore, the high purity of each cell type was confirmed by the boxplot of their ROGUE scores (Figure S1E).

To intuitively characterize the spatial redistribution of cells following RIR, we digitally partitioned both the anterior and posterior segments of the eye by assigning continuous positional values from 1 to 100 along the radial axis (from inner to outer, Figure 1E). Histograms depicting the density distribution of cell types across the anterior and posterior segments were generated (Figure 2C). Our analysis revealed significant spatial redistribution of multiple cell types post‐RIR. Specifically, RGCs, which were tightly clustered in the inner (or medial) region of the posterior segment (corresponding to the GCL layer) in the Sham group, exhibited a pronounced outward shift in their distribution peak after RIR, suggesting RGCs loss and consequent structural compaction of the GCL layer. Microglia in the Sham group were predominantly localized to the outer region of the posterior segment (corresponding to the ONL and RPE layers), likely representing tissue‐resident microglia. Following RIR, a substantial accumulation of microglia was observed in the inner posterior segment (GCL layer), indicating active migration and/or in situ reprogramming. A similar trend of microglial accumulation was noted in the anterior segment post‐RIR. Concurrently, myeloid cells and DCs showed increased aggregation in the inner retinal region (GCL layer) after RIR. In contrast, the spatial distributions of other major cell types remained highly consistent with established anatomical locations: fibroblasts, RPE, and muscle cells were primarily confined to the outer posterior segment (RPE layer); AC, CBC, RBC, and macroglia were enriched in the INL layer; rod and cone photoreceptors were distributed in the intermediate zone of the posterior segment (ONL layer). The aforementioned dynamic cellular changes induced by RIR are further reflected in the differential cellular composition of each ocular region before and after injury (Figures S2, 3).

**Figure 3:**
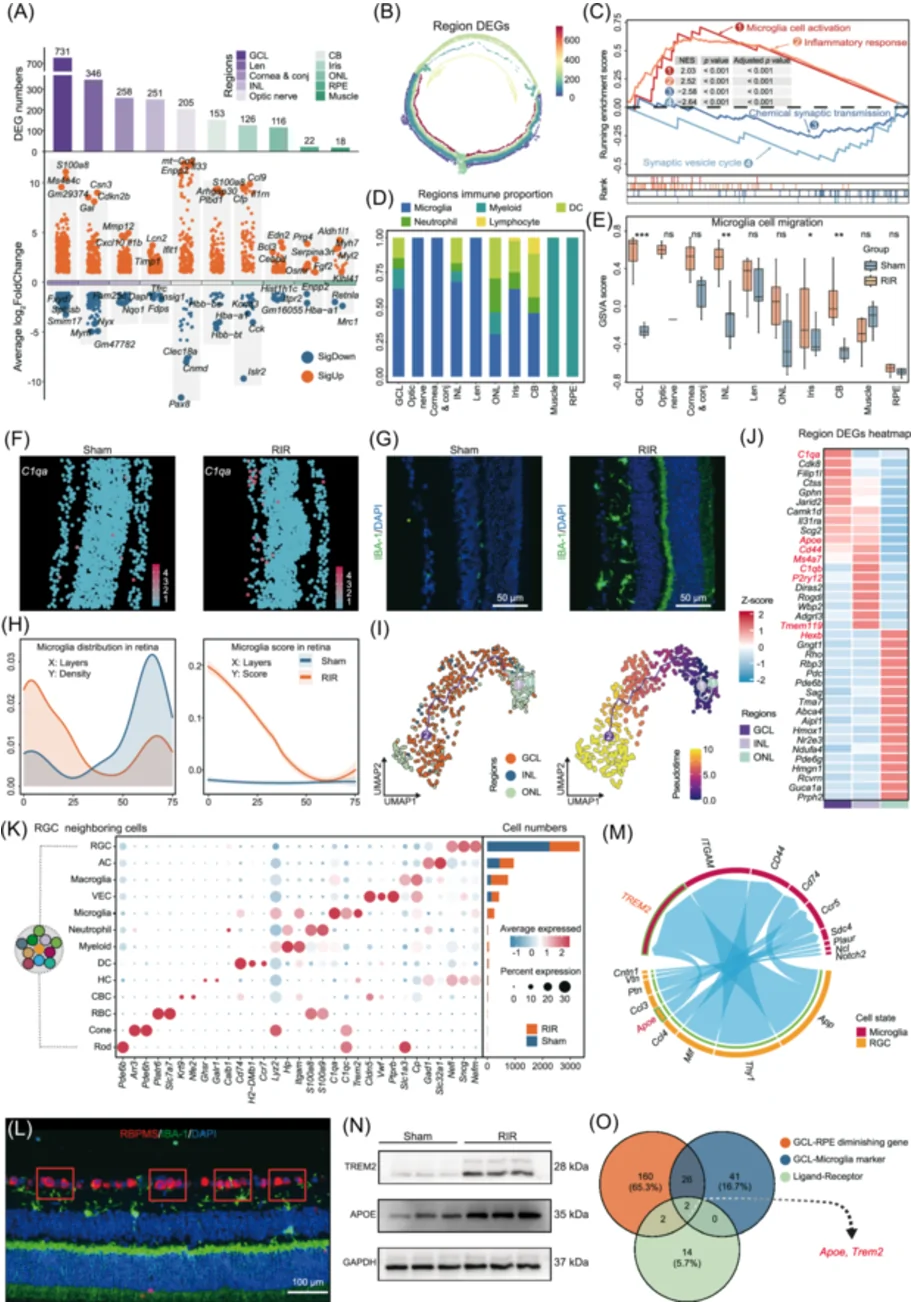
RIR induces molecular topographic changes localized to the GCL microenvironment and microglial spatial redistribution/activation within the inner retinal layers. (A) Differential gene expression analysis revealed both up‐ and down‐regulated genes across the ten regions of the mouse eyeball. Bonferroni correction was applied to the *p‐*value for multiple testing in the DEG analysis. Orange denotes genes with an adjusted *p*‐value < 0.05 and Log_2_FC > 1, while blue indicates those with an adjusted *p*‐value < 0.05 and Log_2_FC < −1. (B) The spatial in situ map shows the number of differentially expressed genes in each region of the Sham group and the RIR group. (C) GSEA showing upregulated immune‐related pathways and downregulated visual function‐related pathways after RIR. The raw *p*‐values were corrected using the Benjamini–Hochberg (BH) method. (D) The stacked bar chart depicts the immune cell composition across various regions following RIR. (E) Boxplot depicting the GSVA scores of the microglia cell migration pathway in ten distinct regions in ocular globes; Statistical significance was assessed using the two‐tailed *t‐*test, and the raw *p* values were corrected using the BH method, with the following notation: ***p < 0.001, **p < 0.01, *p < 0.05; ns indicates not significant. (F) Spatial in situ images showing the expression pattern of *C1qa*, a key marker of microglia. (G) Immunofluorescence images of retinal sections illustrating the tendency of microglia (Iba1, green) to migrate toward the inner retinal layers following RIR. Scale bars, 50 µm. (H) Density distribution plots illustrate the dynamic spatial profiles of microglia along the GCL‐INL‐ONL axis in both Sham and RIR groups (left), and the distribution patterns of microglial signaling pathway activity scores along the same retinal layers (right). (I) UMAP visualization displays the developmental trajectory (left) and pseudotime (right) of microglia along the GCL‐INL‐ONL retinal layers. (J) Heatmap presents the gene expression patterns of microglia across different regions. (K) Dot plot illustrates gene expression patterns of cells neighboring RGCs in the GCL region (left), while a stacked bar chart shows their cellular composition (right). (L) Immunofluorescence of retinal sections demonstrates the spatial co‐localization between microglia and RGCs. (M) Circle plots depicting the receptor‐ligand pairs mediating communication between microglia and RGCs. (N) Western blot analysis of TREM2 and APOE in the Sham and RIR groups. (O) The Venn diagram shows the intersection among GCL‐to‐RPE diminishing genes, GCL microglial marker genes, and receptor‐ligand pair genes between microglia and RGCs. The genes common to all three sets are *Apoe* and *Trem2*. BH, Benjamini–Hochberg; GCL, Ganglion cell layer; GO, Gene Ontology; GSEA, Gene Set Enrichment Analysis; GSVA, Gene Set Variation Analysis; INL, Inner nuclear layer; ONL, Outer nuclear layer; RGCs, retinal ganglion cells; RIR, Retinal ischemia‐reperfusion; RPE, Retinal pigment epithelium.

In summary, RIR injury specifically drives a spatial reorganization of cellular architecture, characterized by RGCs loss (death) and the migration/activation of immune cells, such as microglia, toward the lesion core (the GCL layer).

### RIR injury induces profound disruption of molecular expression landscapes across the anterior segment

We next systematically analyzed the impact of RIR on the trabecular meshwork and corneal regions using ST. Stacked bar plots demonstrated that RIR induced substantial microglial infiltration within the corneal and conjunctival microenvironment (Figure S4A,B). Differentially expressed genes (DEGs) analysis further demonstrated that corneal homeostasis‐associated genes, including *Lypd2*, *Dapl1*, and *Krt12*, were significantly downregulated following RIR, whereas inflammation‐ and stress‐related genes such as *Lyz2*, *Clu*, and *Mmp3* were robustly upregulated (Figure S4C,D). Spatial visualization of corneal marker genes confirmed region‐specific expression patterns of *Krt14*, *Dapl1*, and *Aldh3a1* within the cornea (Figure S4E). Pathway analysis revealed that downregulated pathways following RIR were predominantly enriched in translation and metabolic processes, such as cytoplasmic translation and phenol‐containing compound metabolic process, whereas upregulated pathways were mainly enriched in inflammation‐related pathways, including chemotaxis, taxis, and regulation of immune effector process (Figure S4F).

We next performed a systematic ST analysis of the anterior chamber angle structures. Stacked bar plots revealed pronounced infiltration of inflammation‐associated cells following RIR, with marked increases in neutrophils, lymphocytes, and microglia (Figure S4G). Consistently, DEGs analysis demonstrated significant upregulation of multiple inflammatory genes, including *Spp1*, *Lyz2*, *Serpina3n*, *Cts*b, and *Ctsd*, in the anterior chamber angle after RIR (Figures S4H, 4I). Pathway enrichment analysis revealed that downregulated genes were primarily associated with ocular neural development, such as regulation of neurogenesis, whereas upregulated pathways were mainly related to inflammation, including leukocyte migration, taxis, and chemotaxis (Figure S4J). Spatial visualization of region‐specific markers further showed that *Serpina3n*, *Lyz2*, and *Penk* exhibited distinct spatial enrichment within the anterior chamber angle/corneal regions (Figure S4K). In summary, RIR injury downregulates homeostatic genes, upregulates inflammation/stress genes, and induces microglial infiltration and inflammatory pathway activation in the anterior segment.

**Figure 4:**
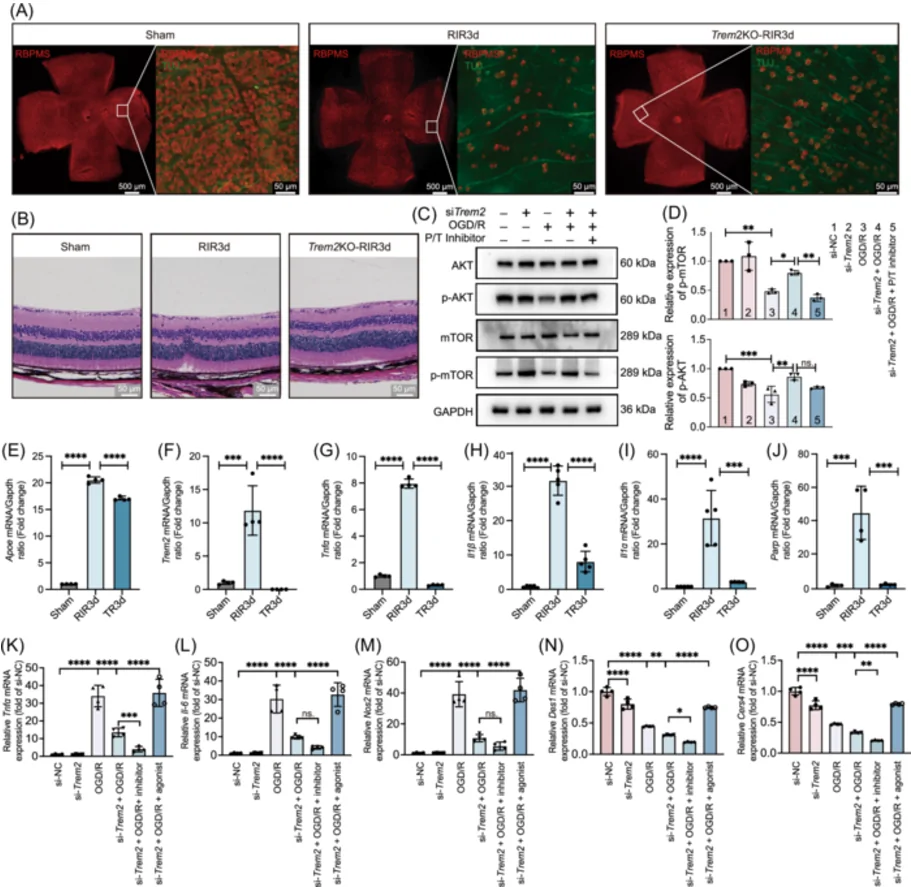
*Trem2* deficiency alleviates RIR‐induced retinal structural damage and inflammatory responses. (A) Morphology of retinal flat mounts (Scale bars, 500 µm) and local enlarged map (Scale bars, 50 µm) from the RIR3d versus *Trem2* KO + RIR3d groups. Quantification of RGCs numbers in retinal flat mounts: RIR3d versus *Trem2* KO + RIR3d groups (adjusted *p* = 0.032; *n* ≥ 6 retinas per group). For each retina, 4–8 symmetrically matched sampling points were analyzed for inter‐group comparison. (B) Comparison retinal H&E section images of the characteristics of Sham, RIR, *Trem2* KO and RIR. Quantification of retinal inner plexiform layer thickness: RIR3d versus *Trem2* KO + RIR3d groups (one‐way ANOVA with Tukey's HSD post hoc test, adjusted *p* = 0.8715; *n* ≥ 6 retinas per group). Scale bars, 50 µm. (C) Western blot analysis of AKT, p‐AKT, mTOR, and p‐mTOR in cellular protein extracts from the 1: control, 2: si‐*Trem2*, 3: OGD/R, 4:si‐*Trem2* + OGD/R, 5: si‐*Trem2* + OGD/R + AKT/mTOR inhibitor. Protein levels were normalized to GAPDH (one‐way ANOVA with Tukey's HSD post hoc test). (D) The bar graphs to the right show the densitometric quantification of p‐AKT and p‐mTOR. Data are presented as mean ± SD (one‐way ANOVA with Tukey's HSD post hoc test). (E–J) RT‐qPCR analysis of inflammatory factors. RT‐qPCR for *Apoe* (E), *Trem2* (F), *TNF‐α* (G), *Il1β* (H), *Il1α* (I), *Parp* (J) in Sham, RIR3d and *Trem2* KO + RIR3d groups. Each dot represents an individual eyeball, and values shown are the mean ± SD; *p* values shown are from one‐way ANOVA with Tukey's HSD post hoc test for each gene. (K–O) RT‐qPCR analysis of the inflammatory factors *Tnfa*, *Nos2* and *Il6* and the metabolic enzymes *Des1, and Cers4* was performed in six groups (control, si‐*Trem2*, OGD/R, si‐*Trem2* + OGD/R, si‐*Trem2* + OGD/R + AKT/mTOR agonist and si‐*Trem2* + OGD/R + AKT/mTOR inhibitor), with each dot representing an individual cell experiment, data presented as mean ± SD, and adjusted *p* values derived from one‐way ANOVA with Tukey's HSD post hoc test. AKT, AKT serine/threonine kinase; GAPDH, Glyceraldehyde‐3‐phosphate dehydrogenase; H&E, Hematoxylin and eosin; mTOR, mechanistic target of rapamycin; OGD/R, Oxygen‐glucose deprivation/reoxygenation; p‐AKT, phosphorylated AKT; p‐mTOR, phosphorylated mTOR; RGCs, retinal ganglion cells; RIR, Retinal ischemia‐reperfusion; RT‐qPCR, Real‐time quantitative polymerase chain reaction; *Trem2* KO, *Trem2* knockout.

### RIR induces spatially restricted inflammation and molecular alterations dominated by microglial activation in the inner retina

DEGs analysis across the 10 spatially defined regions of the mouse eyeball revealed that RIR‐associated transcriptional changes were predominantly enriched in the GCL, Len, and Cornea & conj (Figure 3A,B). Pseudotime analysis incorporating all spatial transcriptomic cells revealed that gene expression could be partitioned into four modules, with pathways mainly enriched in visual perception, translation, and inflammatory response (Figure S5A). Gene set enrichment analysis (GSEA) showed significant upregulation of immune‐related pathways (microglial cell activation and inflammation) and downregulation of eye function‐related pathways (synaptic vesicle cycle and chemical synaptic transmission) after RIR (Figure 3C). Cellular composition analysis further showed that, across multiple regions—including the GCL, INL, ONL, Iris, and CB—immune cell infiltration was dominated by microglia (Figure 3D). Gene set variation analysis (GSVA) demonstrated that microglia cell migration and inflammatory response scores were significantly elevated in the GCL, Cornea & conj, and CB following RIR (Figures 3E, S5B). In addition, complement‐associated proteins (*C5ar1*, *Cd5l*, and *Rab5a*) and chemokines (*Cd74*, *Cxcl10*, and *Cxcl12*) exhibited a progressive inner‐to‐outer decreasing spatial gradient across the GCL‐INL‐ONL‐RPE retinal layers, further indicating that RIR‐induced inflammatory signaling is spatially concentrated within the inner retinal microenvironment (Figure S5H). Together, these findings demonstrate that RIR induces highly spatially specific molecular and inflammatory alterations, positioning the GCL and adjacent inner retinal layers as the principal pathological hubs after ischemia‐reperfusion injury, with microglia serving as potential key effector cells.

Spatial in situ imaging revealed a marked enrichment of the microglial hallmark *C1qa* in the inner retinal layers following RIR, indicating pronounced spatial redistribution of microglia after ischemia‐reperfusion injury (Figure 3F). Immunofluorescence (IF) staining further confirmed this pattern, showing IBA1^+^ microglia migrating toward inner retinal layers after RIR (Figure 3G).

Consistently, spatial density profiling and pathway activity scores demonstrated that microglia preferentially migrated toward the inner retina along the GCL‐INL‐ONL axis, with significant accumulation in the GCL (Figure 3H). Pseudotime trajectory analysis of microglia revealed a continuous progression of cellular states along the GCL‐INL‐ONL axis, with microglia in the GCL exhibiting a more mature and activated phenotype (Figure 3I). Microglia in the GCL upregulate pro‐inflammatory/disease genes (e.g., *C1qa, Apoe, Cd44, Ms4a7*), whereas those in the ONL upregulate photoreceptor/homeostatic genes (e.g., *Hexb, Tmem119, P2ry12, Rho, Pde6b*); INL microglia show a transitional profile linking both states (Figure 3J). Together, these data indicate that RIR induces pronounced microglial spatial relocalization and state transition, positioning the GCL as a central hub of microglial activation.

We next systematically characterized the functional and molecular features of GCL‐associated microglia. GO enrichment analysis revealed that upregulated pathways in GCL microglia were predominantly related to metabolic processes, including lysosome, lipoprotein metabolic process, membrane raft, and phosphatidylserine binding, suggesting substantial lipid metabolic remodeling and membrane reorganization following RIR (Figure S5D). Consistently, AddModuleScore analysis across the GCL‐INL‐ONL regions showed that scores for cell cycle, cell migration, and chemotaxis peaked in the GCL (Figure S5C), reflecting heightened proliferative and migratory activity of microglia in this compartment. To investigate how microglia interact with the RGCs microenvironment, spatial neighborhood analysis revealed that, among cells adjacent to RGCs, immune cells were predominantly microglia (Figure 3K), implicating microglia as key regulators of the RGCs niche. Receptor‐ligand interaction analysis further demonstrated that microglia primarily communicate with RGCs through *Trem2*‐associated signaling, engaging receptors such as *App*, *Thy1*, *Mif*, *Ccl4*, and *Apoe* (Figures 3M, S5F). Interestingly, we found that following injury, interactions between RGCs and surrounding cells are markedly reduced, whereas immune/glial interactions are enhanced, indicating a shift from a neuron‐centered to an immunometabolic state (Figure S5G).

*Apoe* (apolipoprotein E), a central regulator of lipid metabolism, immune modulation, and neural homeostasis, displayed broad expression across multiple retinal cell populations, whereas *Trem*2 was largely restricted to microglia in the retinal single‐cell dataset (Figure S5E). IF of retinal sections shows spatial co‐localization of microglia with RGCs (Figure 3L). Western blot analysis confirmed that protein levels of TREM2 and APOE were significantly elevated in the retina after RIR (Figure 3N). Finally, by intersecting the GCL‐RPE diminishing gene set, GCL‐microglia markers, and ligand‐receptor genes, we identified two core candidates, *Trem2* and *Apoe* (Figure 3O). These lipid‐metabolic molecules may gradually exert regulatory functions along the GCL‐RPE spatial axis, mediating microglial migration and functional transition.

In summary, RIR induces directed microglial migration and state remodeling across retinal layers, leading to their focal accumulation within the GCL. This process is accompanied by *Trem2*‐*Apoe*‐centered lipid metabolic reprogramming, proliferative activation, and enhanced cell‐cell communication, establishing the GCL microenvironment as a principal spatial hub where microglia contribute substantially to inflammatory and metabolic responses following RIR injury.

### *Trem2* deficiency attenuates inflammatory responses and promotes RGCs survival during the early stage

To evaluate the functional role of *Trem2* in RIR, we compared RGCs survival and retinal structure between wild‐type and *Trem2* knockout (*Trem2* KO) mice at 3 days post ‐RIR. Retinal flat‐mount immunostaining revealed a modest but significant preservation of RGCs in *Trem2* KO + RIR retinas compared with RIR controls (Welch's *t*‐test, −81.2 [95%CI −148.0 to −14.4], *p* = 0.032; Figure 4A). Hematoxylin and eosin (H&E) staining further showed a trend toward reduced thinning of the inner plexiform layer in *Trem2* KO + RIR mice, although the difference did not reach statistical significance (Figure 4B). We next examined the effect of *Trem2* deficiency on inflammatory gene expression. Reverse transcription quantitative polymerase chain reaction (RT‐qPCR) analysis demonstrated that the mRNA levels of *Apoe, Trem2, TNF‐α, Il1β, Il1α*, and *Parp* were all significantly elevated in RIR retinas compared with Sham controls. Notably, these increases were markedly attenuated in *Trem2* KO + RIR retinas (Figure 4E–J), indicating that *Trem2* deletion suppresses the RIR‐induced inflammatory response.

To explore the underlying molecular mechanism, we performed in vitro experiments using BV2 microglial cells subjected to oxygen‐glucose deprivation/reoxygenation (OGD/R) with or without si‐*Trem2* knockdown. Western blot analysis showed that OGD/R alone reduced the protein levels of p‐AKT and p‐mTOR, whereas si‐*Trem2* knockdown restored these phosphorylation signals (Figure 4C,D). To confirm that the anti‐inflammatory effect of si‐*Trem2* is mediated through the AKT‐mTOR pathway, we treated si‐*Trem2*‐transfected OGD/R cells with an AKT‐mTOR inhibitor or an agonist. The inhibitor further enhanced the si‐*Trem2*‐mediated suppression of inflammatory cytokines and metabolic enzymes, whereas the agonist reversed these effects (Figure 4K–M). We further examined the expression of sphingolipid metabolic enzymes *Des1* and *Cers4* under the same conditions. A stepwise decreasing trend was observed across the six treatment groups: si‐NC, si‐*Trem2*, OGD/R, si‐*Trem2* + OGD/R, si‐*Trem2* + OGD/R + AKT/mTOR inhibitor, and si‐*Trem2* + OGD/R + AKT/mTOR agonist. Notably, the addition of an AKT/mTOR agonist to the si‐*Trem2* + OGD/R treatment significantly increased *Des1* and *Cers4* expression (Figure 4N,O), confirming that AKT‐mTOR activity positively regulates the expression of sphingolipid metabolic enzymes *Des1* and *Cers4*, and is required for the anti‐inflammatory effect of *Trem2* knockdown in OGD/R‐treated microglia. Collectively, these data indicate that *Trem2* deficiency alleviates RIR‐induced retinal damage and inflammation, with associated down‐regulation of pro‐inflammatory cytokines and sphingolipid metabolic enzymes, suggesting a role for AKT‐mTOR signaling in this process.

These findings suggest that *Trem2* positively regulates the expression of key metabolic enzymes through the AKT‐mTOR signaling pathway, thereby participating in metabolic reprogramming induced by hypoxia‐reperfusion injury.

### *Trem2* deficiency reshapes RIR‐induced metabolic remodeling in the retina

**Figure 5:**
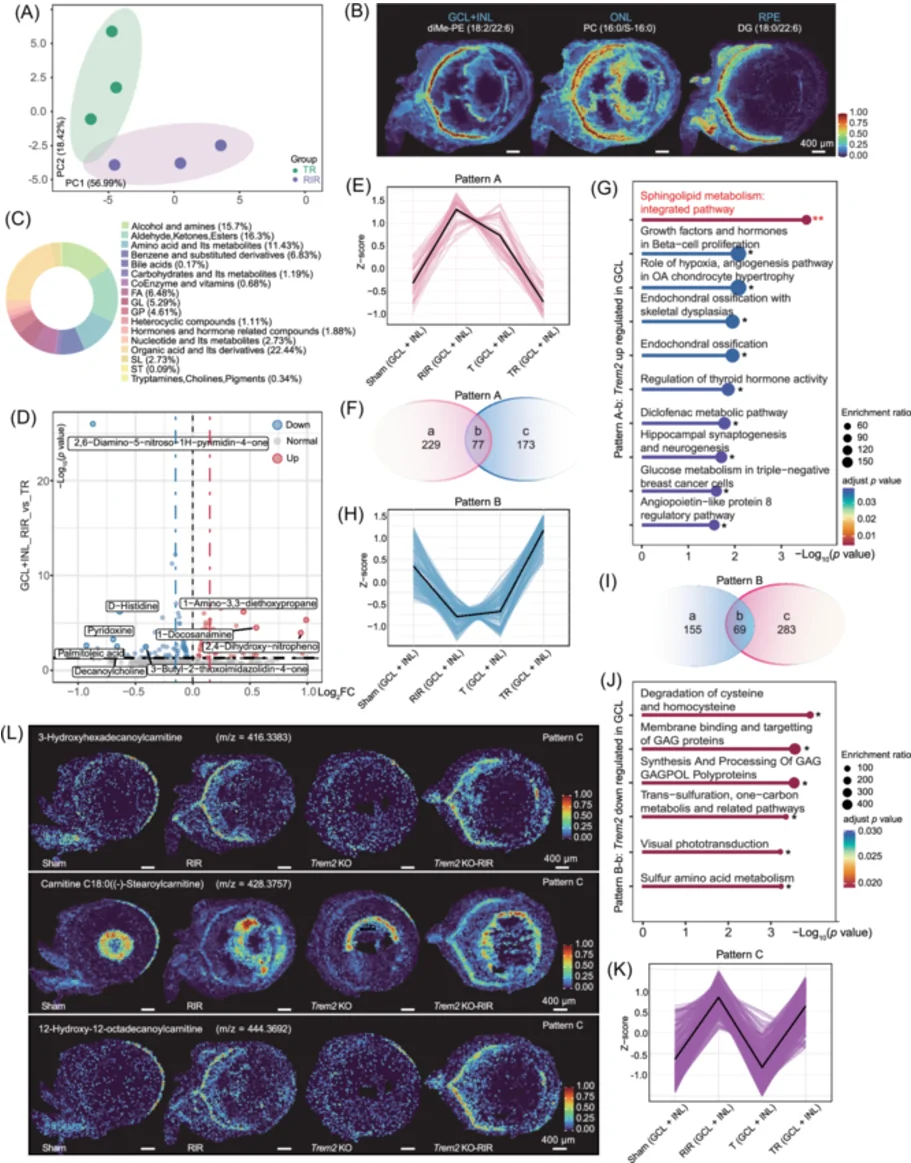
Spatial metabolomics demonstrates the upregulation of sphingolipid metabolism by Trem2. (A) PCA of R (RIR) and TR (*Trem2* KO + RIR) showed significant differences between groups. (B) Retinal layers are defined as GCL + INL, ONL, and RPE based on histological structure. The figure shows the spatial distribution of the top differential metabolite within each layer. (C) Composition of metabolite categories presented in a ring chart. Each color denotes a specific metabolite category, with the segment area indicating its relative abundance. (D) Visualization of differential metabolites in the GCL + INL layer (RIR vs. *Trem2* KO + RIR group) by volcano plot. (E) K‐means clustering of metabolite trends. Pattern A: metabolites upregulated in RIR (vs. Sham) but downregulated in *Trem2* KO + RIR (vs. *Trem2* KO) in the GCL + INL. (F) The Venn diagram depicts overlaps of *Trem2*‐upregulated metabolites in the GCL + INL layer (related to Figure 5E). The intersection (77 metabolites) represents those commonly upregulated in RIR versus Sham (229 metabolites) and downregulated in *Trem2* KO + RIR versus *Trem2* KO (173 metabolites). (G) Functional enrichment analysis was performed on the 77 *Trem2*‐upregulated metabolites, and the raw *p*‐values were corrected using the BH method). (H) Pattern B: metabolites downregulated in RIR (vs. Sham) but upregulated in *Trem2* KO + RIR (vs. *Trem2* KO) in the GCL + INL. (I) The Venn diagram depicts overlaps of *Trem2*‐upregulated metabolites in the GCL + INL layer (related to Figure 5H). The intersection (69 metabolites) represents those commonly upregulated in RIR versus Sham (155 metabolites) and downregulated in *Trem2* KO + RIR versus *Trem2* KO (283 metabolites). (J) Functional enrichment analysis was performed on the 69 *Trem2*‐downregulated metabolites, and the raw *p* values were corrected using the BH method). (K) Pattern C (the most abundant pattern): metabolites upregulated in both RIR (vs. Sham) and *Trem2* KO + RIR (vs. *Trem2* KO) within the GCL + INL. (L) Representative in situ spatial metabolomics images demonstrate the distribution pattern of metabolites belonging to Pattern C. BH, Benjamini–Hochberg; GCL, Ganglion cell layer; INL, Inner nuclear layer; ONL, Outer nuclear layer; RIR, Retinal ischemia‐reperfusion; RPE, Retinal pigment epithelium; *Trem2* KO, *Trem2* knockout.

SM was performed on globe tissues collected from four experimental groups: C (Sham, *n* = 4), R (RIR, *n* = 4), T (*Trem2* KO, *n* = 3), and TR (*Trem2* KO + RIR, *n* = 4) using a *Trem2* KO mouse model subjected to RIR injury. Principal component analysis (PCA) of metabolomic data acquired within the same batch revealed a marked metabolic divergence between the TR and R groups (Figure 5A). Based on the eye globe histological structure, the retina was anatomically segmented into three layers: GCL + INL, ONL, and RPE. The spatial distribution and category composition of 17 representative metabolites were further examined across all groups (Figure 5B,C). Volcano plot analysis showed that, following RIR induction, a subset of metabolites remained significantly upregulated or downregulated under *Trem2*‐deficient conditions, indicating pronounced metabolic remodeling in the GCL + INL layer (Figure 5D). Using K‐means clustering, metabolite dynamics across groups were categorized into three major patterns: (1) Pattern A: Within the combined GCL and INL layer, 77 metabolites were significantly enriched in RIR (vs. Sham) but depleted in *Trem2* KO + RIR (vs. *Trem2* KO). K‐means categorization of “*Trem2*‐upregulated” metabolites reflects the net tissue‐level metabolic effect of *Trem2* in the GCL + INL layer, as measured by bulk SM (Figure 5E,F). Functional enrichment analysis indicated that these *Trem2*‐regulated metabolites were predominantly associated with the sphingolipid metabolism: integrated pathway (Figure 5G). (2) Pattern B: 69 metabolites were downregulated in RIR (vs. Sham) but upregulated in *Trem2* KO + RIR (vs. *Trem2* KO) in the GCL + INL layer (Figure 5H,I), with enrichment mainly linked to the cysteine and homocysteine degradation pathway (Figure 5J). K‐means categorization of “*Trem2*‐downregulated” metabolites reflects the net tissue‐level metabolic effect of *Trem2* in the GCL + INL layer, as measured by bulk SM. (3) Pattern C: metabolites displayed consistent upregulation in both RIR (vs. Sham) and *Trem2* KO + RIR (vs. *Trem2* KO) within the GCL + INL layer, suggesting alterations following RIR modeling with *Trem2*‐independent metabolic remodeling (Figure 5K,L). Representative in situ SM images further confirmed alterations in sphingolipid‐related and pro‐inflammatory metabolites between the RIR and *Trem2*KO + RIR groups in the independent validation dataset (Figure S6).

To assess the clinical relevance, we analyzed multi‐omics data from glaucoma patient aqueous humor. Compared to controls, glaucoma samples showed increased sphingolipid‐related genes, metabolites, and proteins, supporting the TREM2‐sphingolipid axis identified in the RIR model (Figure S7).

### TREM2 binds SPTLC2 and regulates the AKT‐mTOR signaling axis

**Figure 6:**
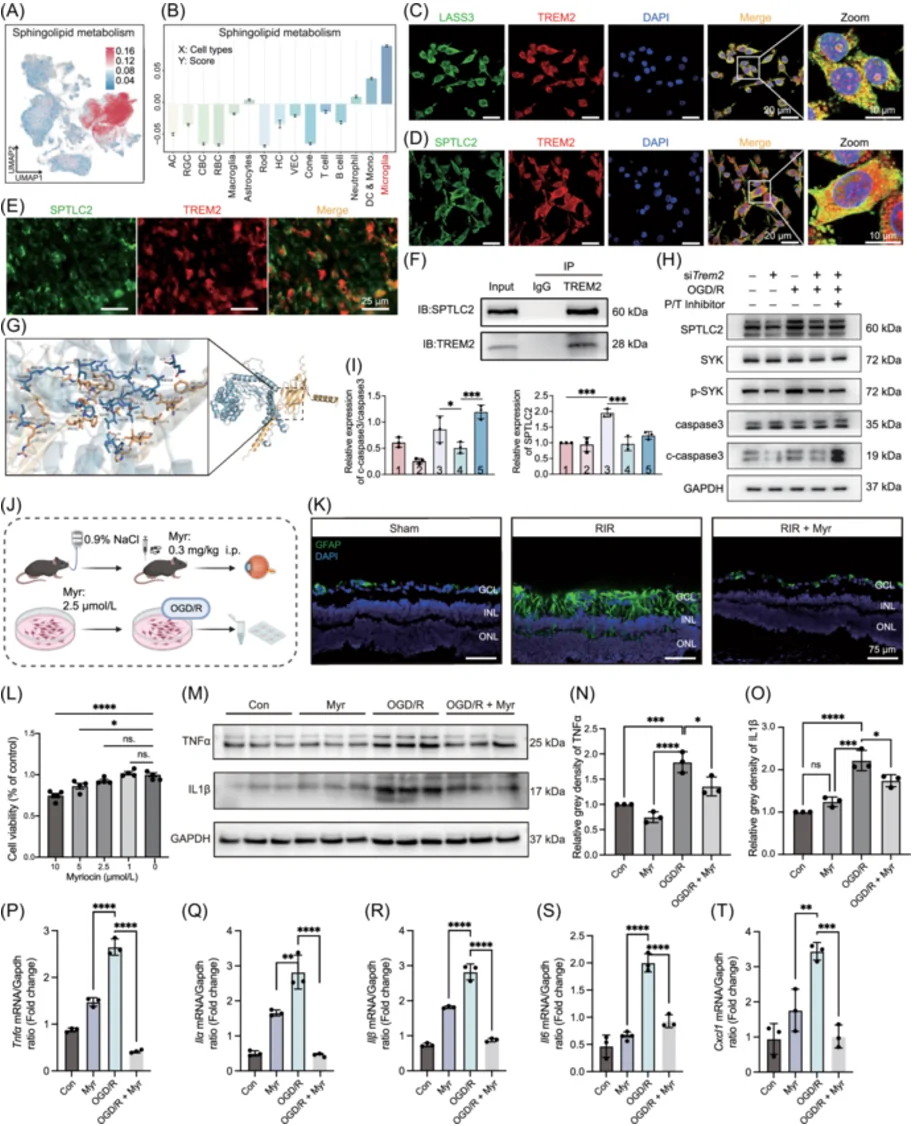
TREM2 binds SPTLC2 and modulates the AKT‐mTOR signaling axis. (A) UMAP plot demonstrating that sphingolipid metabolism is primarily localized in microglia, as validated by single‐cell data from multiple disease models. (B) Among sphingolipid metabolism scores for various retinal cell types, microglia exhibited the highest score. (C) IF staining of LASS3 (green), TREM2 (red), and DAPI (blue) in OGD/R microglia. Co‐localization of LASS3 and TREM2 is shown in yellow. The far‐right panel is a magnified view of the area demarcated by the dotted box in the co‐localization image. Scale bar = 10 µm. (D) IF staining of SPTLC2 (green), TREM2 (red), and DAPI (blue) in OGD/R microglia. Co‐localization of SPTLC2 and TREM2 is shown in yellow. The far‐right panel is a magnified view of the area demarcated by the dotted box in the co‐localization image. Scale bar = 10 µm. (E) IF staining of SPTLC2 (green) and TREM2 (red) in RIR retina. Co‐localization of SPTLC2 and TREM2 is shown in yellow. Scale bar = 25 µm. (F) Verification of the interactions with SPTLC2 and TREM2 in vitro by Co‐IP assay. (G) Molecular docking result between core targets: SPTLC2 and TREM2. (H) Microglia were treated with an AKT‐mTOR pathway inhibitor, with or without OGD/R and/or *Trem2* siRNA, for the indicated times. Protein expression levels were assessed by Western blotting and quantified by densitometry. Representative blots are shown. (I) Quantification of SPTLC2, SYK, p‐SYK, caspase3 and c‐caspase3 in cellular protein extracts from the 1: control, 2: si‐*Trem2*, 3: OGD/R, 4:si‐*Trem2* + OGD/R, 5: si‐*Trem2* + OGD/R + AKT/mTOR inhibitor. Protein levels were normalized to GAPDH or caspase3. Data are presented as mean ± SD. ***p < 0.001, **p < 0.01, *p < 0.05. (J) Schematic diagram summarizing the experimental methodologies for in vivo and in vitro application of Myriocin (Myr). (K) In vivo: Representative retinal immunofluorescence (GFAP, green; DAPI, blue) in Sham, RIR, and RIR + Myr groups. Scale bar = 75 µm. (L) In vitro: Drug toxicity was assessed by CCK‐8 assay following a 24 h treatment of microglia with different concentrations of Myr, using untreated cells as the control. (M–O) In vitro: Western blot analysis of TNF‐α and IL1β in cellular protein extracts from the control, Myr, OGD/R, and OGD/R + Myr‐treated groups. Protein levels were normalized to GAPDH. The bar graphs to the right show the densitometric quantification of TNF‐α (N) and IL1β (O). Data are presented as mean ± SD (analyzed by unpaired, one‐way ANOVA with Tukey's HSD post hoc test). (P–T) In vitro: RT‐qPCR analysis of representative inflammatory factors shows significantly downregulated mRNA levels in the OGD/R + Myriocin group compared to the OGD/R group. Expression levels of *TNF‐α* (P), *Il1α* (Q), *Il1β* (R), *Il6* (S), and *Cxcl1* (T) were quantified. Each dot represents an individual sample. *n* = 3. CCK‐8, Cell Counting Kit‐8; GAPDH, Glyceraldehyde‐3‐phosphate Dehydrogenase; IF, Immunofluorescence; OGD/R, Oxygen and Glucose Deprivation/Reoxygenation; qRT‐PCR, quantitative Real‐Time Polymerase Chain Reaction; RIR, Retinal ischemia‐reperfusion; UMAP, Uniform Manifold Approximation and Projection.

To further elucidate the molecular mechanism by which TREM2 regulates sphingolipid metabolism and microglial inflammatory activation, we first integrated six publicly available scRNA‐seq datasets and annotated microglial cells (Figure S8A–D). These datasets encompass four distinct disease models: autoimmune uveitis (AU), diabetic retinopathy (DR), light damage (LD), and optic nerve crush (ONC). Uniform manifold approximation and projection (UMAP) analysis showed that sphingolipid metabolism‐related genes were predominantly enriched in microglial populations, and metabolic scoring across retinal cell types further confirmed that microglia exhibited the highest sphingolipid metabolic activity (Figure 6A,B). Public scRNA‐seq analysis also showed that *Trem2* is mainly expressed in microglia, and direct co‐localization of *Tmem119* and *Trem2* in retinal tissue further confirmed the microglial identity of *Trem2*‐positive cells (Figure S8E–G). Given that SPTLC2 is a core catalytic subunit of the rate‐limiting enzyme complex initiating de novo sphingolipid biosynthesis, while CERS3 (formerly LASS3) mediates ceramide generation—collectively governing metabolic flux and its downstream effects—we hypothesized that TREM2 directly interacts with these enzymes to regulate sphingolipid metabolism. We therefore used AlphaFold3 to screen for potential binding between TREM2 and several key enzymes, which identified LASS3 and SPTLC2; their direct interaction was subsequently validated using the following methods. IF staining revealed marked co‐localization of TREM2 with both LASS3 and SPTLC2 in OGD/R‐treated microglia (Figure 6C–D). Consistently, spatial co‐localization of TREM2 and SPTLC2 was also observed in RIR retinal tissues (Figure 6E), suggesting that TREM2 may directly participate in the regulation of microglial sphingolipid metabolism. Subsequent co‐immunoprecipitation (Co‐IP) assays validated the physical interaction between TREM2 and SPTLC2 (Figure 6F); under identical experimental conditions, no positive Co‐IP signal was detected between TREM2 and LASS3, indicating binding specificity. Molecular docking analysis further supported a potential binding interface between TREM2 and SPTLC2 (Figure 6G).

Mechanistically, in OGD/R treated microglia, si‑*Trem2* reduced both apoptosis (cleaved caspase‐3/caspase‐3 ratio) and SPTLC2 levels. However, AKT/mTOR inhibitor treatment further increased apoptosis but restored SPTLC2 expression. This uncoupling demonstrates that *Trem2*‑mediated regulation of sphingolipid metabolism occurs through the AKT‑mTOR pathway and is not secondary to changes in microglial cell viability or proliferation (Figure 6H,I). To further verify the functional role of sphingolipid synthesis in microglial inflammatory responses, we performed in vivo and in vitro interventions using Myriocin, an inhibitor of de novo sphingolipid synthesis. In vivo, Myriocin treatment attenuated RIR‐induced GFAP‐positive glial activation in the retina (Figure 6J,K). In vitro cytotoxicity assays defined a safe working concentration range for Myriocin in microglia (Figure 6L). In an OGD/R‐induced microglial inflammatory model, Myriocin treatment significantly reduced the protein levels of TNF‐α and IL1β and downregulated the mRNA expression of inflammatory mediators, including *TNF‐α, Il1α, Il1β, Il6*, and *Cxcl1* (Figure 6M–T).

## DISCUSSION

In this study, we addressed a critical gap in the field by systematically examining how transcriptional and metabolic programs are spatially organized across the entire ocular globe in a 3‐day RIR model. Previous transcriptomic or metabolomic studies have predominantly relied on dissociated cells or homogenized tissues, thereby obscuring region‐specific pathology, cellular interactions, and the spatial origin of metabolic alterations within the retinal microenvironment [16, 28, 29]. By integrating high‐resolution spatial transcriptomics and metabolomics throughout the intact eyeball, we generated a spatially resolved multi‐omics atlas that directly links gene expression programs to local metabolic states. This approach enabled the identification of the GCL + INL as the core pathological region and revealed a spatially confined accumulation of *Trem2* ^*+*^ microglia within this niche. Importantly, these microglial populations exhibited a strong spatial co‐localization with sphingolipid metabolic abnormalities, and knockout of *Trem2* led to marked disruption of sphingolipid metabolism in the inner retina. Mechanistically, TREM2 regulates microglial sphingolipid metabolism by binding to SPTLC2, thereby modulating the AKT‐mTOR signaling axis and inflammatory activation. Furthermore, perturbation of sphingolipid metabolic pathways modulated inflammatory responses, with anti‐inflammatory effects validated in both in vivo and in vitro systems. Collectively, our findings establish a spatial framework in which immune activation and lipid metabolic dysregulation converge within the RGCs microenvironment, highlighting TREM2‐sphingolipid metabolism as a key regulatory axis in retinal neurodegeneration.

RIR injury profoundly reorganizes the retinal microenvironment by shifting pathological regulation from neuron‐centered interactions toward a dominant non‐neuronal immune network [30, 31, 32, 33]. The selective weakening of interactions involving RGCs, together with reinforced communication among non‐neuronal populations, suggests that immune and glial networks become the principal organizers of the pathological niche. Spatial stratification demonstrates that microglial responses differ by retinal layer, reflecting a heterogeneous organization aligned with retinal structure [34]. Microglial migration and redistribution are context‐dependent phenomena across retinal disease models, as retinal microglia demonstrate distinct activation states and spatial patterns under different pathological conditions [35, 36, 37]. For example, Hu et al. observed that activated microglia exhibit a distinct polarization phenotype and accumulate around neovascular lesions in diabetic retinopathy model [35]. Lewis et al. reported that retinal detachment triggered an outward shift of microglia from their usual inner‐retina location [37]. For RIR, previous studies have mainly focused on robust microglial activation in the retina and focal clustering within injury‐adjacent regions [32, 38, 39]. On this background, our spatial analysis further suggests a preferential convergence of microglia toward the inner retinal layers following ischemia‐reperfusion injury, highlighting that the directionality and functional consequences of microglial migration are tightly shaped by the underlying disease context. Together, these observations indicate that the inner retina becomes a focal zone where immune cell redistribution, retinal structure, and neuronal vulnerability intersect, giving rise to a spatially confined pathological ecosystem rather than a diffuse inflammatory response.

In the central nervous system, *Trem2* is widely recognized as a key regulator of microglial lipid sensing, phagocytosis of cellular debris, and metabolic adaptation under stress conditions [40, 41, 42]. Through these functions, *Trem2* supports microglial survival, enhances lipid uptake, and promotes the transition toward disease‐associated microglial states, thereby facilitating debris clearance and tissue remodeling [40, 43, 44]. McQuade et al. demonstrated that *Trem2* deletion reduces microglial survival and impairs phagocytosis of APOE substrates, highlighting its role in metabolic adaptation under pathological stress [45]. Consequently, *Trem2* activity has often been interpreted as neuroprotective or inflammation‐limiting, particularly during early stages of neurodegeneration [46, 47]. However, this interpretation has been challenged by accumulating evidence showing that *Trem2*‐dependent microglia can also adopt inflammation‐associated and lipid‐dysregulated states that sustain microglial activation and contribute to tissue pathology in specific disease contexts [48, 49]. Shi et al. also reported that *Trem2* had dual roles in neuroinflammation, supporting amyloid‐β clearance and tissue protection under normal conditions but driving inflammatory pathology when dysregulated [50]. The “double‐edged” role of *Trem2* is also supported by recent studies of ischemic central nervous system injury. Chen et al. showed that *Trem2* functions differ across stroke models: although *Trem2* is often considered neuroprotective in the conventional middle cerebral artery occlusion model, *Trem2* deficiency reduced infarct size, suppressed inflammation, and preserved neuronal survival in a photothrombotic model that better recapitulates the robust *Trem2* upregulation observed in human stroke [51]. Similarly, our RIR model represents an acute ischemia‐reperfusion injury in which *Trem2* is markedly upregulated in inner retinal microglia, accompanied by lipid metabolic remodeling and inflammatory activation. These findings suggest that *Trem2* does not exert a fixed anti‐inflammatory or neuroprotective function, but instead shapes microglial states in a context‐dependent manner determined by disease type, temporal stage, spatial location, and metabolic environment.

Glaucomatous neurodegeneration extends beyond cell‐autonomous RGCs death and unfolds within a pathological microenvironment in which microglia play an active role [52, 53]. Rather than being uniformly protective or pathogenic, microglial functions are shaped by spatial location, disease stage, and pathological context [54]. Our study provides a spatial and metabolic framework that helps reconcile this longstanding controversy. By integrating spatial transcriptomics and metabolomics across the intact ocular globe, we show that *Trem2* expression defines a microglial population that is not uniformly activated throughout the retina but is selectively enriched within a spatially confined pathological niche in the inner retinal layers. Within this niche, *Trem2* ^+^ microglia are positioned in close proximity to degenerating RGCs and exhibit coordinated activation of sphingolipid metabolism together with inflammatory programs. Importantly, *Trem2* knockout disrupts local sphingolipid metabolic organization and alters inflammatory responses, supporting the idea that *Trem2* actively contributes to the immune‐metabolic state of microglia rather than merely serving as a passive activation marker [55, 56, 57]. Recent single‐cell studies have shown that the microglia program is not uniform. In chronic neurodegeneration, the *Trem2*‐dependent microglia program upregulates lipid metabolism and phagocytosis genes to facilitate debris clearance, whereas acute injury may activate a distinct *Trem2*‐dependent transcriptional program skewed toward cytokine/chemokine production and inflammatory amplification [58]. Our spatial transcriptomic data support this interpretation, showing that in the context of acute retinal ischemia‐reperfusion injury, *Trem2* ^+^ microglia co‐activate metabolic and inflammatory programs, exhibiting features of an acute pro‐inflammatory microglia state. These findings suggest that the functional outcome of *Trem2* signaling is determined by the spatial and metabolic context in which microglia reside. Rather than being intrinsically anti‐inflammatory or pro‐inflammatory, *Trem2* acts as a metabolic regulator that couples lipid remodeling to immune effector functions within a defined structural niche. In the setting of retinal ischemia‐reperfusion injury, this coupling appears to shape a pathological immune‐metabolic‐structural ecosystem that influences the balance between inflammatory damage and tissue adaptation. By resolving *Trem2* function at the level of spatially restricted microglial niches, our study provides a mechanistic explanation for previously conflicting observations and highlights *Trem2*‐dependent lipid metabolism as a critical determinant of microglial behavior in neurodegeneration.

Sphingolipid metabolism represents a particularly relevant metabolic axis in the retina, linking membrane dynamics, inflammatory signaling, and neuronal vulnerability to injury. A study by Simón et al. noted that sphingolipids influence essential retinal processes such as neuronal survival, inflammation, migration, and neovascularization [59, 60]. In the context of retinal ischemia‐reperfusion injury, sphingolipid metabolism is perturbed in a stress‐dependent manner, with ceramide accumulation contributing to RGCs cytotoxicity and inflammatory signaling in retinal degeneration models [61], and our findings further localize these metabolic abnormalities to inner retinal layers, where microglial accumulation and RGCs degeneration coincide. This spatial convergence suggests that sphingolipid pathways participate directly in shaping the local pathological environment rather than reflecting a generalized metabolic disturbance. For microglia, sphingolipids function not only as structural membrane components but also as bioactive mediators that regulate migration, cytokine release, and stress responses [62, 63]. For instance, sphingosine‐1‐phosphate has been shown to induce migration of microglial cells in vitro via activation of volume‐regulated anion channels and purinergic signaling, and accumulation of sphingosine‐1‐phosphate has been linked to microglial activation and pro‐inflammatory responses in vivo [64, 65]. The enrichment of sphingolipid metabolic alterations within microglia‐rich niches provides a mechanistic explanation for how immune cells integrate structural damage cues and translate them into sustained inflammatory outputs [55, 66, 67]. By positioning sphingolipid metabolism downstream of microglial activation yet upstream of inflammatory execution, our findings highlight this pathway as a key regulatory layer through which microglia influence retinal pathology.

The spatial confinement of sphingolipid metabolic dysregulation to microglia‐enriched inner retinal niches suggests that therapeutic efficacy may depend not only on pathway selection but also on cellular and spatial precision. Rather than globally suppressing inflammation or lipid metabolism across the retina, interventions that selectively modulate microglial sphingolipid programs within defined pathological niches may achieve more favorable outcomes. The observation that perturbation of sphingolipid metabolism attenuates inflammatory responses both in vivo and in vitro supports the feasibility of targeting this axis, while also emphasizing that the functional consequences of such modulation are context‐dependent. Importantly, the spatial heterogeneity revealed in this study implies that metabolic interventions may exert divergent effects depending on disease stage, microglial state, and retinal layer involvement. Extending spatially resolved metabolic analyses to longitudinal settings and complementary disease models will be essential to determine how durable and generalizable this regulatory axis is [68, 69, 70]. Together, these considerations position sphingolipid metabolism not as a universal target, but as a spatially and temporally constrained lever for modulating retinal immune pathology.

### Limitations of the study

Several limitations should be acknowledged. First, although our multi‐omics and imaging data strongly implicate microglia as a major contributor to the observed sphingolipid metabolic changes, direct lipid‐level validation in purified microglial populations was not performed; contributions from other cell types (e.g., Müller glia or stressed RGCs) cannot be formally excluded. Matrix‐assisted laser desorption/ionization mass spectrometry imaging (MALDI‐MSI) reflects relative ion abundance rather than absolute quantification; direct lipid flux measurements in microglia are warranted in future studies. Second, while pharmacological inhibition of sphingolipid synthesis with myriocin attenuated microglial inflammation, we did not directly assess whether myriocin treatment normalizes the specific sphingolipid species identified by MALDI‐MSI (e.g., ceramides, sphingomyelins). Third, our causal inferences rely on whole‐body *Trem2* knockout mice and siRNA‐mediated knockdown in microglial cells; microglia‐specific *Trem2* conditional knockout was not used. Fourth, formal rescue experiments with exogenous ceramides were not performed due to technical challenges in ceramide delivery and acyl‐chain specificity. Finally, this study focused on the acute phase (72 h) of RIR injury; the role of the TREM2‐sphingolipid axis in chronic or repetitive injury models remains to be investigated.

## CONCLUSION

By integrating ST, SM, and scRNA‐seq multi‐dimensional analyses, this study suggests that RIR is not solely driven by intrinsic neuronal damage but is amplified and shaped by spatially defined immunometabolic remodeling in which *Trem2* ^*+*^ microglia play an important role. These findings deepen our understanding of the cellular mechanisms underlying glaucoma‐associated neurodegeneration and identify *Trem2* and its associated sphingolipid metabolic pathways as promising targets for precise immunometabolic intervention and therapeutic development.

## METHODS

### Mice

Experimental mice included wild‐type male C57BL/6J (6–8 weeks, 18–25 g; Medical Laboratory Animal Center of Guangzhou, China) and *Trem2*KO mice with controls (Cyagen, China; Catalog C001207). Mice were housed in an SPF facility under controlled temperature (21 ± 1°C), humidity (60 ± 5%), and a 12‐h light/dark cycle.

### RIR modeling and animal grouping

The RIR surgery was performed according to our established protocol [71]. After surgical induction of RIR injury, mice received a 7‐day treatment regimen of Myriocin (Thermozymocidin, CAS 35891‐70‐4, APExBIO) administered intraperitoneal injection (i.p.) at 0.3 mg/kg per day. Control animals in the Sham and RIR groups were given an equivalent volume of 0.9% saline solution during the same period.

For ST analysis, mice were divided into two groups: Sham and RIR. Independent biological replicates consisted of individual animals: *n* = 5 mice (Sham) and *n* = 6 mice (RIR). From each animal, one coronal whole‐eye section passing through the optic nerve was collected for Stereo‐seq library preparation. All data were generated on the same platform, using identical sequencing chemistry and spatial resolution (resolution of 500 nm). Sequencing was performed in two batches: an initial discovery batch containing *n* = 2 animals per group, and a second validation batch comprising the remaining *n* = 3 (Sham) and *n* = 4 (RIR). All comparisons treated the individual animal, not the tissue section, as the independent experimental unit.

For spatial metabolomic analysis, four groups were analyzed: Sham (*n* = 4 mice), RIR (*n* = 4 mice), *Trem2*KO (*n* = 3 mice), and *Trem2* KO + RIR (*n* = 4 mice). From each animal, one coronal whole‐eye section passing through the optic nerve was subjected to MALDI‐MSI. Biological replicates refer to independent mice. Data acquisition was performed in two batches: an initial discovery batch comprising *n* = 1 per group, followed by a second validation batch containing the remaining *n* = 3 per group (*n* = 2 for *Trem2*KO). In the Sham group, the validation batch was analyzed using air‐flow‐assisted desorption electrospray ionization (AFADESI) mass spectrometry imaging as a complementary ionization method, while all other samples were analyzed by MALDI‐MSI. Key metabolic findings identified in the discovery batch were confirmed in the independent validation batch.

### Histological examination

Histological analysis was performed at 3 days post‐operation following our established protocol [71]. For quantification, four retinal cross‐sections within a 1 mm radius of the optic nerve head were analyzed per eye, with inner plexiform layer thickness determined using Fiji (ImageJ 1.54p, Wayne Rasband and contributors National Institutes of Health, USA http://imagej.org).

### Immunofluorescence staining

Detailed procedures for immunofluorescence staining of retinal cryosections and whole mounts, including tissue fixation, primary and secondary antibody incubation, DAPI nuclear staining, mounting, and image acquisition, were performed as described in our previous study [71]. The following primary antibodies were used in this study: TUJ1 (1:500, 801202, Biolegend), RBPMS (1:500, GTX118619, GeneTex), and GFAP (1:300, 12389, Cell Signaling Technology). For cell IF staining, cells were seeded onto confocal dishes, fixed with 4% paraformaldehyde (10 min), permeabilized with 0.3% Triton X‐100 (10 min), and blocked with goat serum (1 h). Primary antibodies against LASS3 (1:200, Abmart, #PC24624), SPTLC2 (1:200, Proteintech, China, #51012‐2‐AP), and TREM2 (1:200, Abmart, #M079355s) were incubated overnight at 4°C. After washing, cells were incubated with Alexa Fluor 488/594‐conjugated secondary antibodies (1:500) and DAPI (1:1000). Images were acquired using an FV3000 fluorescence microscope (Olympus). Co‐localization analysis was performed with ImageJ.

### OGD/R modeling and cellular interventions

The BV2 microglial cell line was obtained from Zhong Qiao Xin Zhou Biotechnology. PI3K/mTOR inhibitor‐11 (100 nM, GLPBIO, GC69709) was added to the culture medium 1 h or Myriocin (Thermozymocidin, CAS 35891‐70‐4, APExBIO) was added to the culture medium 24 h prior to OGD and maintained throughout the experiment until cell harvesting. To recapitulate in vivo RIR injury, BV2 cells were incubated in glucose‐ and serum‐free medium inside a hypoxic chamber (5% CO_2_, 95% N_2_) at 37°C for 3 h. Subsequently, cells were returned to normoxic conditions (37°C, 5% CO_2_) and normal culture medium (DMEM supplemented with 10% fetal bovine serum and 4.9 g/L glucose) for 24 h of reperfusion prior to harvest.

*Trem2* (NM_031254) knockdown (si‐*Trem2*) was performed using siRNA, and negative control RNA was generated by Sango Biotech (Shanghai, China). The knockdown efficiency was validated by Western blotting, and the selected siRNA oligonucleotide sequences are as follows:

**Table:** 

si‐*Trem2*	5'−3'	Forward	GGCUGAGGUCCUGCAGAAATT
	3'−5'	Reverse	UUUCUGCAGGACCUCAGCCTT

### Western blot and co‐immunoprecipitation (Co‐IP) analysis

Protein extraction from retinal tissue and cells was performed using a commercial protein extraction kit (KeyGEN, KGB5303‐100). Subsequently, protein separation was carried out by electrophoresis on 10%–15% gradient polyacrylamide gels following standard protocols. The expression levels of target proteins were normalized to GAPDH from the same sample, with its value set as 1.0 for quantification. Primary antibodies and their dilutions used were as follows: TNF‐α (1:1000, CST, USA, #11948), IL1β (1:1000, CST, USA, #12242), and APOE (1:1000, CST, USA, #49285), SPTLC2 (1:1000, Proteintech, China, #51012‐2‐AP), and TREM2 (1:1000, Abmart, China, #M079355s), SYK (1:1000, CST, USA, #13198T), p‐SYK (1:1000, CST, USA, #44170T), Caspase3 (1:1000, CST, USA, #9662), c‐Caspase3 (1:1000, CST, USA, #9664).

Co‐IP was performed using retinal tissue homogenates lysed in immunoprecipitation lysis buffer (Beyotime, China, #P0013). After centrifugation, the resulting lysates were mixed with the appropriate primary antibodies and rotated overnight at 4°C, followed by a 4 h incubation at 4°C with Pierce™ Protein A/G Agarose. The immunoprecipitated target proteins were subsequently eluted and detected by Western blotting. The primary antibodies used in this study were: anti‐SPTLC2 (Proteintech, China, 1:1000, #51012‐2‐AP), anti‐Flag (Merck, USA, 1:2500, #F1804), and anti‐TREM2 (Abmart, China, 1:1000, #M079355s).

### RT‐qPCR

Total RNA was isolated from microglia or retina tissue using Color Reverse Transcription Kit (A0010CGQ, ESscience) in accordance with the manufacturer's instructions. RNA concentration and purity were determined with a NanoDrop 2000 spectrophotometer (Thermo Scientific). Quantitative real‐time PCR was carried out on Light Cycler 480 Real‐Time PCR System with software version LCS480 1.5.1.62 using 2× Color SYBR Green qPCR Master Mix (A0012‐R2, ESscience). The sequences of all gene‐specific primers used in this study are provided in Table S4 (Supporting Information).

### Molecular docking

Protein sequences were downloaded from UniProt. The entries used for each species are Q99NH8 (TREM2, Mouse) and P97363 (SPTLC2, Mouse). Protein‐protein docking was performed using the online server HDOCK (http://hdock.phys.hust.edu.cn/) under default parameters. Hydrogen bonds, hydrophobic contacts, and other non‐covalent interactions between TREM2 and SPTLC2 were analyzed using PyMOL (The PyMOL Molecular Graphics System, Version 2.5).

### Stereo‐seq spatial transcriptomics

Stereo‐seq spatial transcriptomic profiling was performed by STOmics [72] in accordance with previously published protocols. Briefly, 10‐μm‐thick frozen tissue sections were mounted onto Stereo‐seq chips and fixed with methanol, followed by tissue imaging to preserve morphological information. The tissue sections were then permeabilized, allowing RNA molecules to be captured by spatially barcoded probes on the chip and subjected to in situ reverse transcription. The resulting cDNA was released from the tissue, purified, and amplified. Subsequently, the amplified cDNA was fragmented using Tn5 transposase and used for sequencing library construction. The final libraries were subjected to high‐throughput sequencing on the MGI DNBSEQ‐T1 platform.

### Processing of spatial sequencing data

Raw spatial sequencing data were processed using the SAW (STOmics Analysis Workflow, https://github.com/BGIResearch/SAW) pipeline. Quality control was performed on the paired‐end sequencing reads as follows: (1) The Coordinate Identifier (CID) sequences were aligned against the chip's barcode library to extract read pairs with valid CIDs; (2) The CID was decoded and converted into spatial coordinate information, which was integrated into the identifier of Read2; (3) Read2 underwent quality filtering to obtain clean reads.

The quality‐controlled reads were then aligned to the reference genome (GRCm38) using the STAR aligner. Uniquely mapped reads were assigned to their corresponding gene annotation regions. Reads with identical unique molecular identifiers (UMIs) at the same spatial coordinate and gene locus were collapsed to remove PCR duplicates.

Finally, based on the location of each DNA nanoball (DNB) on the chip, the Bin1, which is the minimal spatial resolution unit, a spatially‐resolved gene expression count matrix (spots × genes) was generated. This matrix served as the foundation for all subsequent spatial analyses.

### Alignment of sequencing results with the paired staining image

The Stereo‐seq chip contains periodic reference lines that run along its entire 1 cm length, with a width of 1.5 µm. These reference lines are clearly visible in the staining images. To effectively utilize the staining images (single‐stranded DNA images) for subsequent analysis, alignment between the sequencing results and the staining images is necessary. During the alignment procedure, the sequencing results are first converted into RNA signal expression images (RNA images). Reference lines are then marked on these RNA images using image analysis software such as ImageJ or Photoshop. Subsequently, the RNA images and single‐stranded DNA images are aligned precisely based on these reference lines to ensure an alignment error of less than 1 µm (equivalent to 2 points, given a point‐to‐point distance of 500 nm on the chip). This alignment process was performed by one bioinformatician and independently verified by two additional bioinformaticians to ensure double‐checking.

### Cell segmentation of spatial transcriptomic data

After alignment, to achieve spatial single‐cell resolution, cell segmentation was performed using the Stain‐segmentation method in Spateo [73]. Nuclei were segmented from the single‐stranded DNA images via the watershed algorithm, and each nucleus was subsequently expanded. A uniform expansion criterion was applied to all cells, with the maximum expansion distance set to no more than 5 µm to prevent overlap or excessive expansion relative to nuclear size. Each cell was then assigned a unique identifier. Finally, RNA signals from both the nuclear and expanded cytoplasmic regions were aggregated as the transcriptional profile for that cell. Only cells expressing more than 30 genes were retained for downstream analysis.

### Processing, dimensionality reduction, clustering, and annotation of public scRNA‐seq datasets

Public mouse ocular single‐cell datasets were obtained from ArrayExpress (E‑MTAB‑8119), NCBI GEO (GSE146186, GSE182419, GSE182477, GSE182582, GSE182583, and selected GSM samples), and the GSA of the China National GeneBank (PRJCA008174). Using Seurat v5.3.0 in R 4.4.2, we applied SCTransform (default parameters) for normalization, followed by PCA (120 PCs) and Harmony dimensionality reduction. An SNN graph was built with 60 neighbors and 100 PCs, then clustered at resolution 1.2 to yield 47 clusters. UMAP was generated for visualization. DEGs per cluster were identified via MAST test (FindAllMarkers, min.pct = 0.1, logfc.threshold = 1) with Bonferroni correction (*p* < 0.01). Clusters were annotated into 27 cell types based on established marker genes and literature. The curated data were integrated with spatial transcriptomics for downstream analyses.

We collected mouse retinal single‐cell data from four retinal disease models (AU/DR/LD/ONC) from GEO, covering six datasets (GSE132229, GSE191260, GSE199792, GSE205123, GSE126783, GSE199317; total 172,480 cells, 25,757 genes). Using Seurat, SCTransform normalized the data. PCA was run on the top 4000 highly variable genes, followed by Harmony batch correction. An SNN graph was built with 80 neighbors and 120 PCs, then clustered at resolution 1.4 to obtain 57 clusters. UMAP was used for visualization. DEGs were identified with the same MAST criteria (Bonferroni‐corrected *p* < 0.01). The 57 clusters were annotated into 16 cell types using known marker genes and literature.

### Identification and validation of gene sets associated with different regions

Spatial co‐expression analysis was performed using Hotspot (version 0.9) [74], using 5000 highly expressed genes as input to Hotspot, and each co‐expressed gene module was defined as a group of genes with high spatial expression correlation. H&E staining was used to identify different regions of the mouse eyeball. The genes with the highest frequency were sorted into different region‐related gene sets. The gene set was validated by calculating the enrichment score of all cells in all mouse eyes, including the Sham group and the RIR group. The collected region‐related gene sets were used to perform functional analysis of DEGs using the R package clusterProfiler (version 4.6.2) [75]. For each GO term gene set in the biological process field, we used the “enrichGO” functions to perform over‐representation enrichment analysis, respectively. The adjusted *p*‐value < 0.05 was used as the threshold to screen out significantly enriched GO term.

### Dividing mouse eyeball into 10 regions

The mouse eyeball comprises multiple distinct anatomical regions. Leveraging the spatial resolution of transcriptomic data, we systematically segmented the eyeball by integrating region‐specific gene signatures with histological staining patterns. First, using the curated region‐specific marker gene sets collected in Section 2.11, we calculated signature scores for each spatial cell utilizing the AddModuleScore() function in Seurat. These scores provided a transcriptional basis for regional identification. This computational approach was complemented by histological annotation. Based on registered immunofluorescence staining images and corresponding H&E staining, mask images for distinct anatomical layers, such as the GCL and RPE, were generated using ImageJ. Following this protocol, we further refined the segmentation to precisely delineate 10 anatomical compartments: the GCL, INL, ONL, RPE, CB, Iris, len, Optic nerve, Cornea & conj, and Muscle.

### Annotation of spatial transcriptomic data

To deconvolve the cellular composition within the mouse eyeball, we performed cell type annotation on segmented spatial transcriptomics data using the Robust Cell Type Decomposition method (RCTD, version 2.2.1; https://github.com/dmcable/spacexr) [76]. A pre‐processed single‐cell RNA‐seq reference dataset (described in Section 2.10) was loaded using the Reference() function. Spatial transcriptomic data were prepared by extracting spatial coordinates, raw expression matrices, and per‐cell nUMI counts via the SpatialRNA() function. Cell type deconvolution and annotation were then carried out with the run.RCTD() function in doublet mode.

To enhance annotation accuracy, tissue regions were analyzed separately: CB & Iris, cornea & conjunctiva (Cornea & conj), retina, RPE, and len were each subjected to independent RCTD‐based annotation. For the optic nerve and muscle regions, cell types were identified through dimensionality reduction, clustering, and manual annotation based on established marker genes.

### Digitization of the anterior and posterior regions

To enable an intuitive characterization of cellular distribution and gene expression patterns, digital partitioning of the mouse eyeball was performed using the dd.digitize() function in the Spateo software package [73]. This method digitally segmented the eyeball into an anterior region‐comprising the CB, Iris, Cornea & conj, and Len and a posterior region, which included the GCL, INL, ONL, RPE, and Muscle. The underlying algorithm is based on the principle of potential energy theory from physics, and its execution required the manual input of four boundary coordinates defining the ocular wall. The interactive coordinate selection process was conducted following the official online tutorial (https://spateo-release.readthedocs.io/en/latest/tutorials/notebooks/5_cluster_digitization/6_layer_column_digitization.html). Subsequently, each cell was assigned a corresponding digital positional score on a continuous scale from 1 to 100.

### GO analysis

GO functional enrichment analysis was performed in the R environment using the clusterProfiler package [75]. Gene annotation information was obtained from the corresponding species‐specific annotation databases (e.g., org.Mm.eg.db). Statistical significance of GO term enrichment was assessed using the hypergeometric test, and *p* values were adjusted for multiple comparisons using the Benjamini–Hochberg method. For each cell type, 5–10 disease‐associated and significantly enriched GO pathways were selected for visualization.

### GSEA analysis

We used the Findmarker function in Seurat to perform DEGs analysis on the GCL region of the mouse eyeball between the Sham and RIR groups, and selected genes with an absolute Log2FC value greater than 1 and an adjusted *p*‐value less than 0.05 as DEGs. The genes were sorted by Log2FC size, and the gene set selected was the KEGG and GO: BP geneset in the MSigDB (https://www.gsea-msigdb.org/gsea/msigdb/). The fgsea (https://github.com/alserglab/fgsea/) function in the software fgsea was used for KEGG and GO GSEA (Gene Set Enrichment Analysis) analysis.

### Analysis of cell trajectory

To delineate the continuous changes in cell states along biological processes, a pseudotime trajectory analysis was performed. Expression matrices of target cell subsets (e.g., microglia) were extracted from the ganglion cell layer, inner nuclear layer, and outer nuclear layer of the mouse retina. Subsequently, single‐cell trajectories were constructed using the Monocle 2 software package (version 2.30.1) [77]. A Monocle object was created with the new_cell_data_set() function, and cell trajectories were inferred using the learn_graph() function.

### Cell–cell communication analysis

To decipher the cell‐cell interaction network within the retinal microenvironment, a systematic cell communication analysis was performed using the CellChat software (version 1.5.0) [78], based on the normalized single‐cell and spatial transcriptomic gene expression matrices, calculates intercellular communication networks and predicts major signaling pathways; only receptors and ligands expressed in at least 10% of specific cells are further analyzed.

### Spatial metabolomic

Spatial metabolomic profiling was performed by Wuhan Metware Biotechnology Co., Ltd. using MALDI‐MSI. Frozen tissue samples were cryosectioned at 10–12 μm thickness, mounted onto indium tin oxide (ITO)‐coated conductive slides, and vacuum‐dried. A 2,5‐dihydroxybenzoic acid (DHB) matrix was uniformly applied using an automated sprayer to ensure homogeneous matrix crystallization.

MALDI‐MSI data were acquired on a timsTOF flex mass spectrometer (Bruker Daltonics) operated in positive ion mode, with a mass range of m/z 50–1300 and a spatial resolution of 20 μm. Spectra from each pixel were generated by averaging multiple laser shots under constant laser energy. Raw spectral data were normalized using root mean square normalization. Metabolite identities were assigned based on accurate mass matching and further confirmed by tandem mass spectrometry (MS/MS) when applicable. Spatial metabolite distributions were subsequently aligned with adjacent H&E‐stained tissue sections for anatomical interpretation.

### SM data analysis

Unsupervised PCA was performed in R (prcomp function) on unit variance‐scaled data. Hierarchical cluster analysis and Pearson correlation analysis were then conducted using the ComplexHeatmap package, with results visualized as heatmaps (dendrograms included; metabolite intensities color‐scaled). For two‐group comparisons, differential metabolites were filtered by |log_2_FC| ≥ 0.26 and identified by matching to public databases such as KEGG Compound. Significantly regulated pathways were further analyzed via metabolite set enrichment analysis, with statistical significance assessed using the hypergeometric test.

### Multi‐omics data collection and processing

We downloaded a published bulk RNA‐seq public dataset (GSE101727) from the GEO database, containing aqueous humor samples from 10 patients with primary open‐angle glaucoma (POAG) and 10 control subjects. The data were normalized using the log‐normalize method, and GSVA analysis was performed based on sphingolipid metabolism‐related gene sets from MSigDB (Table S3). Metabolomics and proteomics data from the same study (PMC11627470) were obtained from its supplementary materials, and an additional independent proteomics dataset (POAG vs. cataract controls) was collected from the iProX database (IPX0002299000). All data processing was carried out using R software (version 4.4.2), extracting sphingolipid metabolism‐related metabolites and proteins. The Wilcoxon rank‐sum test was used for all comparisons between the POAG and control groups.

### Pseudobulk generation and batch correction

In this study, spatial transcriptomic data from two independent batches were used. For each sample, pseudobulk count profiles were generated by aggregating the raw UMI counts of all cells within that sample using the sum function. To eliminate batch effects, the pseudobulk count matrix was corrected using the ComBat_seq() function from the sva R package. After batch correction, the resulting count matrix was normalized using the log‐normalization method. The normalized pseudobulk expression matrix was then used for downstream analyses.

### Statistical analysis

For in vitro experiments, each condition was tested in at least three independent biological replicates. For in vivo experiments, group sizes are indicated in the corresponding figure legends and represent individual animals. Data are shown as mean ± SD and were processed with GraphPad Prism version 8.0.2 and R (version 4.3.2) for statistical analysis and graphing. Significance was assessed by unpaired two‐tailed *t*‐test, Wilcoxon rank‐sum test, or one‐way ANOVA with Bonferroni correction, where appropriate. The threshold for statistical significance was set at *p* < 0.05, with markers defined as: ns (not significant), *p < 0.05, **p < 0.01, ***p < 0.001, and ****p < 0.0001.

## AUTHOR CONTRIBUTIONS

**Yunhong Shi**: Conceptualization; methodology; data curation; validation; investigation; writing—original draft; writing—review and editing; project administration; visualization. **Jinpei Lin**: Software; data curation; supervision; formal analysis; validation; visualization; writing—review and editing; methodology. **Yi Wu**: Writing—original draft; writing—review and editing. **Shengsong Xu**: Writing—review and editing. **Naiyuan Zhang**: Formal analysis; validation; writing—review and editing; visualization. **Yiji Pan**: Writing—review and editing; validation. **Anli Ren**: Writing—review and editing. **Ying Fang**: Resources. **Yu Huang**: Writing—review and editing. **Xiayin Zhang**: Writing—review and editing. **Zhuoting Zhu**: Writing—review and editing. **Yehong Zhuo**: Writing—review and editing; supervision; resources; conceptualization; funding acquisition. **Honghua Yu**: Writing—review and editing; supervision; resources; conceptualization; funding acquisition.

## CONFLICT OF INTEREST STATEMENT

The authors declare no conflicts of interest.

## ETHICS STATEMENT

All experiments complied with institutional ethical guidelines and were specifically approved by the Animal Care and Use Committee of Guangdong Provincial People's Hospital (No. KY2025‐1314‐01) and the Committee of the Zhongshan Ophthalmic Center (No. 02021080).

## Supporting information


**Figure S1:** Single‐cell reference atlas and spatial transcriptomic characterization of the mouse eyeball.
**Figure S2:** Spatially resolved single‐cell atlas of the ocular globe in Sham and RIR.
**Figure S3:** Spatial cellular composition and UMAP dimensionality reduction of distinct regions in the Sham and RIR groups.
**Figure S4:** Molecular and spatial responses of the anterior segment to RIR injury.
**Figure S5:** Molecular signatures and spatial distribution of the inflammatory microenvironment in RIR injury.
**Figure S6:** *In situ* distribution of sphingolipid and pro‐inflammatory metabolites.
**Figure S7:** Multi‐omics validation of sphingolipid metabolic status in human aqueous humor.
**Figure S8:** Microglial profiling across four mouse retinal disease models.

**Table S1:** Single‑cell reference atlas and spatial transcriptomic characterization of the mouse eye.
**Table S2:** Abbreviations.
**Table S3:** Sphingolipid metabolism‐related gene set.
**Table S4:** The primers used for the amplification of the target genes.

## Data Availability

The data that support the findings of this study are available from the corresponding author upon reasonable request. Data generated during this study have been deposited in the Genome Sequence Archive in BIG Data Center, Beijing Institute of Genomics (BIG, https://ngdc.cncb.ac.cn/gsa/), Chinese Academy of Sciences, under the Project Accession No. OMIX011996 https://ngdc.cncb.ac.cn/omix/release/OMIX011996 and PRJCA041282 https://ngdc.cncb.ac.cn/bioproject/browse/PRJCA041282. The data and scripts used are saved in GitHub: https://github.com/pienapple1/eyeball_STSM. Supplementary materials (figures, tables, graphical abstract, slides, videos, Chinese translated version, and updated materials) may be found in the online DOI or iMeta Science (http://www.imeta.science).
