# Supplementary material for: Spatial multi‐omics unveils sphingolipid metabolic reprogramming within the retinal pathological niche
Source: Imeta. 2026 Aug 20;5(4):e70163. doi: 10.1002/imt2.70163 (PMC13494123; doi:10.1002/imt2.70163)
Supplement: Supplementary file 1 [file IMT2-5-e70163-s001.docx]

**Supporting information to:**

**Spatial multi-omics unveils sphingolipid metabolic reprogramming within the retinal pathological niche**

**Running title:** Sphingolipid metabolic reprogramming within retina

Yunhong Shi^1^^,2#^, Jinpei Lin^3,4#^, Yi Wu^1#^, Shengsong Xu^2,5#^, Naiyuan Zhang^1#^, Yiji Pan^1^, Anli Ren^1^, Ying Fang^1^, Yu Huang^6^, Xiayin Zhang^7^, Zhuoting Zhu^8^, Yehong Zhuo^2^*, Honghua Yu^9^*

^1^Guangdong Eye Institute, Department of Ophthalmology, Guangdong Provincial People’s Hospital (Guangdong Academy of Medical Sciences), Southern Medical University, Guangzhou 510080, China.

^2^State Key Laboratory of Ophthalmology, Zhongshan Ophthalmic Center, Sun Yat-sen University, Guangdong Provincial Key Laboratory of Ophthalmology Visual Science, Guangzhou 510060, China.

^3^College of Life Sciences, University of Chinese Academy of Sciences, Beijing 100049, China.

^4^Analysis Center of Agrobiology and Environmental Sciences, Zhejiang University, Hangzhou 310058, China.

^5^Shenzhen Eye Hospital, Shenzhen Eye Medical Center, Southern Medical University, Shenzhen 518040, China.

^6^Department of Eye and Vision Sciences, Institute of Life Course and Medical Sciences, University of Liverpool, L69 7ZX, United Kingdom.

^7^Singapore Eye Research Institute, Singapore National Eye Center, 168751, Singapore.

^8^Centre for Eye Research Australia, University of Melbourne; Department of Surgery (Ophthalmology), University of Melbourne, Melbourne, VIC 3010, Australia.

^9^Joint Shantou International Eye Center of Shantou University and The Chinese University of Hong Kong, Shantou, 515000, China.

^#^These authors contributed equally: Yunhong Shi, Jinpei Lin, Yi Wu, Shengsong Xu, Naiyuan Zhang.

*Correspondence to: yuhonghua@stu.edu.cn (Honghua Yu); [zhuoyh@mail.sysu.edu.cn](mailto:zhuoyh@mail.sysu.edu.cn) (Yehong Zhuo)

**Figure S1 Single-cell reference atlas and spatial transcriptomic characterization of the mouse eyeball.** (A) 12 publicly available single-cell transcriptomic datasets from diverse tissues and experimental platforms, establishing a reference atlas for spatially resolved cellular characterization. (B) Dot plot showing the expression of marker genes across 27 single-cell types. Dot size reflects the percentage of cells expressing the gene, and color intensity indicates the average expression level. (C) UMAP of integrated spatial transcriptomic cells. Left: Sham; right: RIR. (D) UMAP of spatial transcriptomic samples (top) and ten ocular regions (bottom). (E) Boxplots showing cell purity for each cell type by ROGUE index in Spatial transcriptomics. The closer the value is to 1, the higher the purity. RIR, Retinal ischemia-reperfusion; Prolif. cell, Proliferation Cell; Sch. cell, Schwann cell; Fibro., Fibroblast; Lymph., Lymphocyte; Conj, Conjunctival Cell; Cornea & Conj, Cornea and Conjunctiva; CB, Ciliary Body; GCL, Ganglion cell layer; INL, Inner nuclear layer; ONL, Outer nuclear layer; RPE, Retinal pigment epithelium; Muscle, Extraocular Muscle.

**Figure S2 Spatially resolved single-cell atlas of the ocular globe in Sham and RIR.** (A) Sham group. Left: whole-eye spatial transcriptomic view (scale bar, 0.5 mm). Right: three magnified views of selected regions: retina, CB, and cornea (scale bar, 0.1 mm). (B) RIR group. Same layout as (A). Cell type color codes are consistent across all subpanels (A and B). The color legend for the 29 cell types is provided at the bottom left corner of the Figure S2. RIR, Retinal ischemia-reperfusion; CB, Ciliary body.

**Figure S3 Spatial cellular composition and UMAP dimensionality reduction of distinct regions in the Sham and RIR groups.** Single-cell resolution spatial transcriptomic atlas of the mouse whole eyeball. (A-F) UMAP visualizations show cell type distributions across in ocular globes: Retina (GCL, INL and ONL) (A), Cornea & conj (B), CB & Iris (C), RPE & Muscle (D), Len (E), and Optic nerve (F). Corresponding cell type proportion plots across samples are also shown. For each panel, the color-coded legend is located below the UMAP plot. Cornea & conj, Cornea and Conjunctiva; CB, Ciliary Body; GCL, Ganglion cell layer ; INL, Inner nuclear layer; ONL, Outer nuclear layer; RPE, Retinal pigment epithelium; Muscle, Extraocular Muscle; UMAP, Uniform Manifold Approximation and

**Figure S4 Molecular and spatial responses of the anterior segment to RIR injury.** (A) Stacked bar chart of cell types in corneal and conjunctiva samples. (B) Spatial transcriptomics reveals the *in situ* distribution of major cell types in the Cornea & conj. (C) Volcano plot of differentially expressed genes between the Sham and RIR groups in corneal and conjunctiva samples, and the raw *p* values were corrected using the BH method. (D) Heatmap showing differentially expressed genes in the corneal region between the Sham and RIR groups. (E) Spatial expression patterns of markers of the corneal region (*Krt14*, *Dapl1*, and *Aldh3a1*) in the transcriptomic dataset. (F) Gene set enrichment analysis identifying biological processes associated with downregulation (left) and upregulation (right) in the RIR group compared to the Sham group in corneal and conjunctiva samples. (G) Stacked bar chart of cell types in Anterior chamber angle structures (including the iridotrabecular meshwork). (H) Volcano plot of differentially expressed genes between the Sham and RIR groups in CB and Iris; Bonferroni correction was applied to the *p* value for multiple testing in the DEG analysis. (I) Heatmap showing differentially expressed genes in the CB and Iris region between the Sham and RIR groups. (J) Gene set enrichment analysis identifying biological processes associated with downregulation (left) and upregulation (right) in the RIR group compared to the Sham group in CB and Iris. (K) Spatial expression patterns of regional marker genes (*Serpina3n*, *Lyz2*, and *Penk*) in the transcriptomic dataset. RIR, Retinal ischemia-reperfusion; GO, Gene Ontology; Z-score, Standardized expression Z-score; Cornea & conj, Cornea and Conjunctiva; VS., versus; -Log*p*, Negative Log-transformed *p* value; adj., adjust; Log_2_FC, Log_2_Fold Change; *p*.adjust, Adjusted *p* value; BH, Benjamini-Hochberg.

**Figure S5 Molecular signatures and spatial distribution of the inflammatory microenvironment in RIR injury.** (A) Pseudotime trajectory analysis of gene expression dynamics related to inflammatory and developmental processes. Left: Heatmap showing gene expression across pseudotime-ordered cells. Middle: Key genes corresponding to each expression pattern. Right: Significantly enriched GO biological processes associated with each pattern. (B) Boxplot depicting the GSVA scores of the Inflammatory response pathway in ten distinct regions in ocular globes; Statistical significance was assessed using the two-tailed *t*-test, and the raw *p* values were corrected using the BH method, with the following notation: *** *p* < 0.001, ** *p* < 0.01, * *p* < 0.05; ns indicates not significant. (C) Boxplot display AddModuleScore activity estimates for cellular processes-including Cell cycle, Cell migration, and Cell chemotaxis-in microglia within the GCL-ONL-INL regions; Statistical significance was assessed using the Wilcoxon rank-sum test, and the raw *p* values were corrected using the BH method), with the following notation: *** *p* < 0.001, ** *p* < 0.01, * *p* < 0.05; ns indicates not significant. (D) Gene Ontology terms of upregulated biological process in the GCL region. (E) Left: Barplot showing expression levels of *Apoe* and *Trem2* across cell types in the spatial transcriptomic data. Right: Spatial *in situ* maps showing the expression distribution of *Apoe* (top) and *Trem2* (bottom) in the Sham and RIR groups. (F) Circle plot showing the interactions between retinal microglia and other cells via the APOE signaling pathway in the RIR group. (G) The bar graph illustrates the comparative number of receptor-ligand pairs across various retinal cell types before and after modeling. (H) The spatial distribution patterns of complement proteins and chemokine-related genes (or molecules) across the GCL-INL-ONL retinal layers. RIR, Retinal ischemia-reperfusion; GCL, Ganglion cell layer ; INL, Inner nuclear layer; ONL, Outer nuclear layer; RPE, Retinal pigment epithelium ; GSVA, Gene Set Variation Analysis; GO, Gene Ontology; Z-score, Standardized expression Z-score; -Log*p*, Negative Log-transformed *p* value; LR, Ligand-Receptor; BH, Benjamini-Hochberg.

**Figure S6 *In situ* distribution of sphingolipid and pro-inflammatory metabolites.** (A) *In situ* validation of sphingolipid-related metabolites in TR versus RIR groups based on secondary sequencing. (B) *In situ* validation of pro-inflammatory metabolites in TR versus RIR groups based on secondary sequencing. RIR, Retinal ischemia-reperfusion; TR, *Trem2*KO + RIR.

**Figure S7 Multi-omics validation of sphingolipid metabolic status in human aqueous humor.** (A) Bulk RNA-seq analysis showing significantly upregulated expression of key sphingolipid metabolism-related genes (*SPTLC1*, *UGCG*, *SPHK2*, *GLB1*, *ACER2*) in POAG samples compared to controls. (B) GSVA enrichment scores demonstrating enhanced activity of sphingolipid metabolism and related pathways in POAG patients. (C) Metabolomics profiling showing increased levels of sphingolipid-related metabolites in POAG samples. (D) Proteomics analysis identifying significantly upregulated proteins associated with sphingolipid metabolism in POAG clinical specimens. Statistical comparisons for all panels (A-D) were performed using the Wilcoxon rank-sum test, and the raw *p* values were corrected using the BH method, with significance indicated as: *** *p* < 0.001, ** *p* < 0.01, * *p* < 0.05; ns indicates not significant. POAG, Primary Open-Angle Glaucoma; Cont., Control; GSVA, Gene Set Variation Analysis; Exp. level, Expression level; meta. level, Metabolite level; BH, Benjamini-Hochberg.

**Figure S8 **Microglial profiling across four mouse retinal disease models.**** (A) UMAP visualization of single-cell transcriptomic data, colored by cell type. (B) UMAP plot showing cell distribution across disease statuses (AU, DR, LD and ONC). (C) Feature plots of homeostatic/resident microglial markers (*P2ry12,* *Tmem119*, *Siglech*). (D) Feature plots of infiltrating monocyte/macrophage markers (*Ccr2*, *Plac8*, *Ifitm2*). (E) Feature plot showing *Trem2* expression levels across retinal cells. (F) Dot plot illustrating average expression of key myeloid markers across myeloid cell subsets. (G) Immunofluorescence staining validating co-expression of TMEM119 and TREM2 in retinal microglia. scRNA-seq, Single-Cell RNA Sequencing; AU, Autoimmune Uveitis; DR, Diabetic Retinopathy; LD, Light Damage; ONC, Optic Nerve Crush; Mφ/Mono, Macrophage/Monocyte; Mono., Monocyte.
